# PPAR gamma 2 Prevents Lipotoxicity by Controlling Adipose Tissue Expandability and Peripheral Lipid Metabolism

**DOI:** 10.1371/journal.pgen.0030064

**Published:** 2007-04-27

**Authors:** Gema Medina-Gomez, Sarah L Gray, Laxman Yetukuri, Kenju Shimomura, Sam Virtue, Mark Campbell, R. Keira Curtis, Mercedes Jimenez-Linan, Margaret Blount, Giles S. H Yeo, Miguel Lopez, Tuulikki Seppänen-Laakso, Frances M Ashcroft, Matej Orešič, Antonio Vidal-Puig

**Affiliations:** 1 Department of Clinical Biochemistry, Histopathology, University of Cambridge/Addenbrooke's Hospital, Cambridge, United Kingdom; 2 Technical Research Centre of Finland (VTT), Espoo, Finland; 3 University Laboratory of Physiology, University of Oxford, Oxford, United Kingdom; Stanford University School of Medicine, United States of America

## Abstract

Peroxisome proliferator activated receptor gamma 2 (PPARg2) is the nutritionally regulated isoform of PPARg. Ablation of PPARg2 in the ob/ob background, PPARg2^−/−^ Lep^ob^/Lep^ob^ (POKO mouse), resulted in decreased fat mass, severe insulin resistance, β-cell failure, and dyslipidaemia. Our results indicate that the PPARg2 isoform plays an important role, mediating adipose tissue expansion in response to positive energy balance. Lipidomic analyses suggest that PPARg2 plays an important antilipotoxic role when induced ectopically in liver and muscle by facilitating deposition of fat as relatively harmless triacylglycerol species and thus preventing accumulation of reactive lipid species. Our data also indicate that PPARg2 may be required for the β-cell hypertrophic adaptive response to insulin resistance. In summary, the PPARg2 isoform prevents lipotoxicity by (a) promoting adipose tissue expansion, (b) increasing the lipid-buffering capacity of peripheral organs, and (c) facilitating the adaptive proliferative response of β-cells to insulin resistance.

## Author Summary

It is known that obesity is linked to type 2 diabetes, however how obesity causes insulin resistance and diabetes is not well understood. Some extremely obese people are not diabetic, while other less obese people develop severe insulin resistance and diabetes. We believe diabetes occurs when adipose tissue becomes “full,” and fat overflows into other organs such as liver, pancreas, and muscle, causing insulin resistance and diabetes. Peroxisome proliferator activated receptor gamma (PPARg) is essential for the development of adipose tissue and control of insulin sensitivity. PPARg2 is the isoform of PPARg regulated by nutrition. Here we investigate the role of PPARg2 under conditions of excess nutrients by removing the PPARg2 isoform in genetically obese mice, the POKO mouse. We report that removing PPARg2 decreases adipose tissue's capacity to expand and prevents the mouse from making as much fat as a normal obese mouse, despite eating similarly. Our studies suggest that PPARg plays an important antitoxic role when it is induced in liver, muscle, and beta cells by facilitating deposition of fat as relatively harmless lipids and thus prevents accumulation of toxic lipid species. We also show that PPARg2 may be involved in the adaptive response of beta cells to insulin resistance.

## Introduction

An adipocentric view of the Metabolic Syndrome (MS) considers obesity as the major factor leading to insulin resistance in peripheral metabolic tissues. However, the link between obesity and insulin resistance is complex, as indicated by the fact that some extremely obese people are glucose tolerant, while others with a mild degree of obesity develop severe insulin resistance and diabetes. This suggests that the absolute amount of fat stored may not be the most important factor determining the relationship between obesity and insulin resistance. Recent work showing the complexity of the molecular mechanisms controlling adipogenesis [1,2] suggests that adipose tissue expandability may be an important factor linking obesity, insulin resistance, and associated comorbidities.

There are two mechanisms that have been proposed to explain how expansion of the adipose tissue stores affects insulin sensitivity. One mechanism suggests that increased adiposity induces a chronic inflammatory state characterized by increased cytokine production by adipocytes and/or from macrophages infiltrating adipose tissue. Cytokines produced by these adipocytes or macrophages may directly antagonise insulin signalling [3,4]. A second nonexclusive hypothesis is lipotoxicity. The lipotoxic hypothesis states that if the amount of fuel entering a tissue exceeds its oxidative or storage capacity, toxic metabolites that inhibit insulin action are formed [5–8]. Of particular relevance to this article, lipid metabolites, such as ceramides and diacylglycerol (DAG) or reactive oxygen species generated from hyperactive oxidative pathways, have been shown to inhibit insulin signalling and to induce apoptosis [9–11].

The nuclear receptor peroxisome proliferator activated receptor gamma (PPARg) is critically required for adipogenesis and insulin sensitivity [12–15]. There are two PPARg isoforms, PPARg1 and PPARg2. PPARg1 is expressed in many tissues and cell types, including white and brown adipose tissue, skeletal muscle, liver, pancreatic β-cells, macrophages, colon, bone, and placenta [16]. Under physiological conditions, expression of PPARg2, the other splice variant, is restricted to white and brown adipose tissue [16,17]. In adipose tissue PPARg is the key regulator of adipogenesis. PPARg2 is the more adipogenic PPARg isoform in vitro, it is also the isoform regulated transcriptionally by nutrition [17–20]. Although under physiological conditions expression of PPARg2 is limited to adipose tissues, we have shown that PPARg2 is ectopically induced in liver and skeletal muscle in response to overnutrition or genetic obesity [2,18]. De novo expression of PPARg2 in liver and muscle in obesity suggests that PPARg2 may have a role in insulin resistance and lipotoxicity in these tissues. Little in vivo research into the metabolic roles for the specific isoforms of PPARg has been carried out, with the studies so far focusing almost exclusively on adipose tissue [2,13,21,22]. PPARg (both isoforms) deletions have been generated in most major metabolic tissues. Liver-specific deletion of both PPARg isoforms caused an impairment in insulin sensitivity, particularly when challenged by different genetic backgrounds (lipoatrophic or leptin-deficiency) [23,24]. The effect of ablating both PPARg isoforms in muscle produced controversial results, with two groups reporting different effects on insulin sensitivity [25,26]. The role of PPARg in pancreatic β-cells is unclear, primarily due to its low expression under physiological conditions [27–29] and secondly because ablation of both PPARg isoforms in β-cells did not result in a metabolic phenotype. However PPARg may play a role in β-cell hyperplasia in response to insulin resistance, an idea supported by the fact that mice that lack PPARg in β-cells do not expand their β-cells mass in response to a high-fat diet [30]. More recently, it has been shown that heterozygous PPARg-deficient mice develop impaired insulin secretion, which is associated with increased islet triacylglycerol (TAG) content [31].

Here we investigate the physiological relevance of PPARg2 under conditions of positive energy balance by ablating PPARg2 in ob/ob mice. We use a new approach that integrates traditional physiological phenotyping with advanced lipidomic technology and transcriptomics. Our results indicate that in the context of positive energy balance, the absence of PPARg2 results in a major metabolic failure. Furthermore, we provide evidence that control of adipose tissue expansion by PPARg2 may be an important variable linking positive energy balance to its metabolic complications including insulin resistance, β-cell failure, and dyslipidaemia. Similarly, our lipidomic results indicate that induction of PPARg2 in nonadipose tissues should be considered as a physiological adaptation that prevents the toxic effects produced by excess nutrients. This antilipotoxic effect of PPARg2 is achieved by increasing the lipid-buffering capacity of peripheral organs and facilitating β-cell hyperplasia in response to insulin resistance.

## Results

### Ablation of PPARg2 in Ob/Ob Mice (POKO Mouse) Prevents Adipose Tissue Expansion in Response to Positive Energy Balance

PPARg2^−/−^ Lep^ob^/Lep^ob^ mice with genetic ablation of the PPARg2 isoform on the obese hyperphagic ob/ob background (POKO) were generated. Matings of PPARg2^+/−^ Lep^ob^/Lep^+^ mice followed the expected Mendelian distribution (Fisher's test = 0.074 and 0.135 for males and females, respectively). PPARg1 gene expression in white adipose tissue (WAT) from five-week-old POKO mice was similar to PPARg2 KO mice and was not significantly different from wild-type (WT) mice (Figure S1).

**Figure 1:**
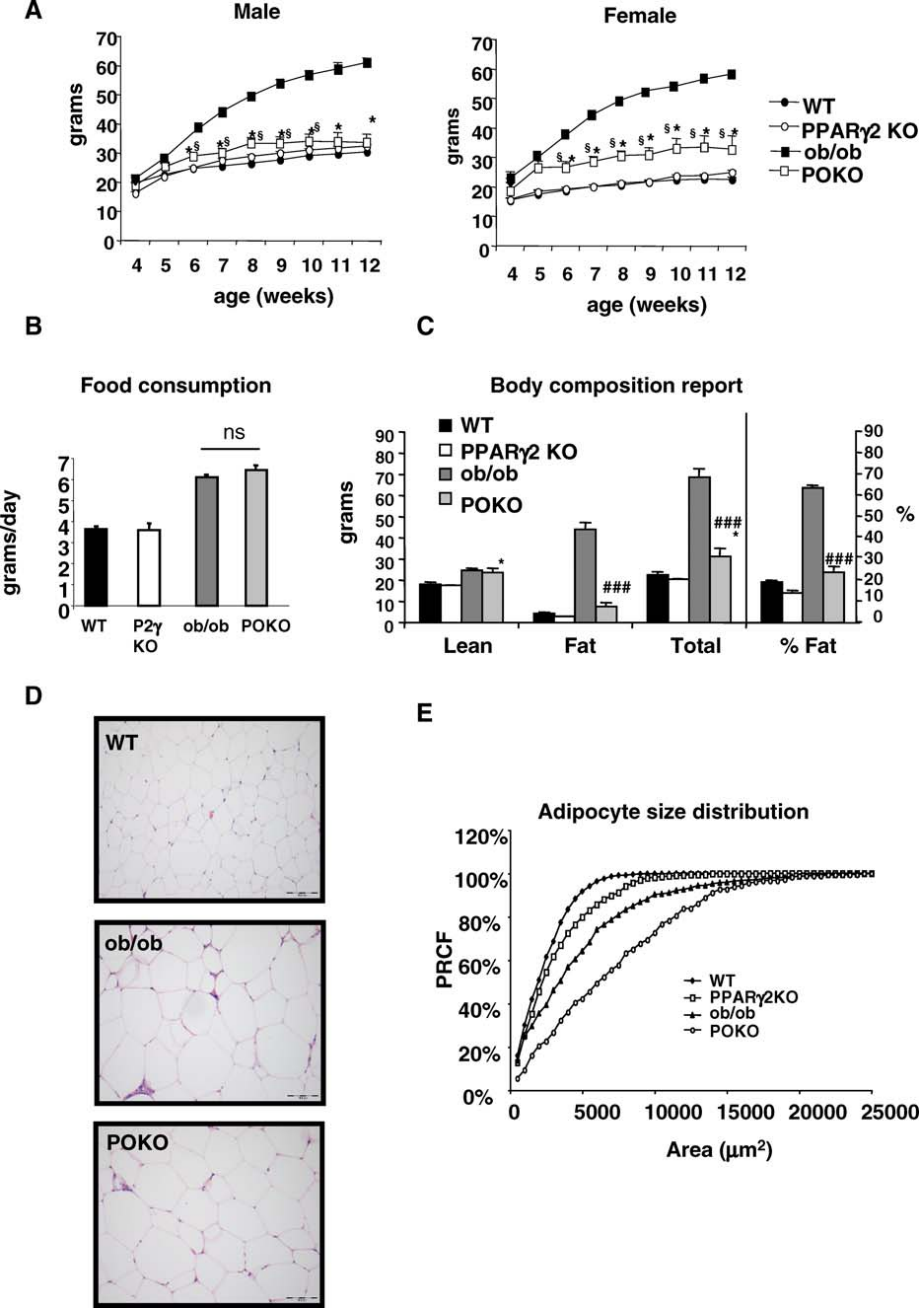
Physiological Characterisation of POKO Mouse (A) Body weights (black circles, WT; black squares, ob/ob; white circles, PPARg2 KO; white squares, POKO) are shown for males (left) or females (right) (*n* = 5–12). *, *p* < 0.05 POKO versus ob/ob and §, *p* < 0.01 POKO versus WT. (B) Food intake from 20-wk-old female mice (*n* = 4) is shown. (C) Body composition analysis from 20-wk-old females is shown: WT, ob/ob, PPARg2 KO, and POKO mice fed chow diet mice (*n* = 4–7). *, *p* < 0.05 POKO versus WT and ###, *p* < 0.001 POKO versus ob/ob. (D) Haematoxylin and eosin (H and E)-stained sections (10×) from epididymal WAT from 16-wk-old male WT, ob/ob, and POKO mice. (E) Percent relative cumulative frequency analysis (PRCF) from epididymal WAT adipocytes from 16-wk-old male WT, ob/ob, PPARg2 KO, and POKO mice. (*n* = 4–5).

Figure 1A shows growth curves for male and female mice of four genotypes (WT, PPARg2 KO, ob/ob, and POKO mice) over a 12-week period. At birth, the body weight of male and female POKO mice was indistinguishable from other genotypes (unpublished data). The ob/ob mice quickly became heavier than their WT littermates, with significantly elevated body weight by four and six weeks of age in female and male mice, respectively. However, the POKO mice did not become obese, and their body weight remained close to WT and PPARg2 KO body weights mice during the 12-week study.

POKO mice were as hyperphagic (Figure 1B) as the ob/ob mice but drank far more water compared with ob/ob littermates (81.85 ± 15.14 versus 9.05 ± 2.32 ml/70 h, *p* < 0.01, female POKO versus ob/ob, *n* = 4 at 20 wk) (Figure S2A). Dual-energy X-ray absorptiometry analysis at 20 wk (Figure 1C) confirmed that female POKO mice had slightly increased fat content (4%) compared to WT and PPARg2 KO mice, but significantly reduced fat mass compared to the 40% increase observed in ob/ob mice. At the age of 20 wk, POKO and ob/ob mice had a trend to a decreased total locomotor activity during dark and light cycles compared with the WT and PPARg2 KO mice over the 72-h period. However POKO had similar total locomotor activity compared with ob/ob mice (Figure S2B).

At six weeks of age, female POKO mice consumed a similar amount of oxygen as ob/ob mice (vO_2_ = 25.06 ± 0.89 versus 23.10 ± 0.99 ml/kg bodyweight ^0.75^/min, *p* = 0.07 POKO versus ob/ob, *n* = 6–8) showing a lower respiratory exchange ratio (0.916 ± 0.011 versus 0.952 ± 0.007, *p* = 0.01, female POKO versus ob/ob) in the fed state, but similar respiratory exchange ratio in the fasted state (0.73 ± 0.014 versus 0.75 ± 0.018, *p*-value = 0.59 POKO versus ob/ob mice). Water intake was already significantly increased in POKO compared to ob/ob mice (13.59 ± 1.88 versus 8.15 ± 0.89 ml/d, *p*-value < 0.05, POKO versus ob/ob). Furthermore, levels of glucose in urine were higher in POKO mice compared with ob/ob mice (403.4 ± 49.2 versus 34.13 ± 13.5 mMol/l, POKO versus ob/ob mice, *p*-value = 0.001), showing an energy loss of 15.43 ± 3.06 kJ/d through urine compared with 0.70 ± 0.19 kJ/d in ob/ob mice. At this age, POKO mice showed similar locomotor activity compared with the ob/ob mice during the day, but increased locomotor activity during the night (Figure S2C).

Histomorphometric analysis of adipose tissue from 16-wk-old male mice revealed that POKO mice had fewer small adipocytes than the ob/ob mice (Figure 1D and 1E). This analysis of adipocyte size suggests that ablation of PPARg2 in the ob/ob background impairs the potential for adipocyte recruitment.

### Early Insulin Resistance in POKO Mice Independent of Body Weight

**Figure 2:**
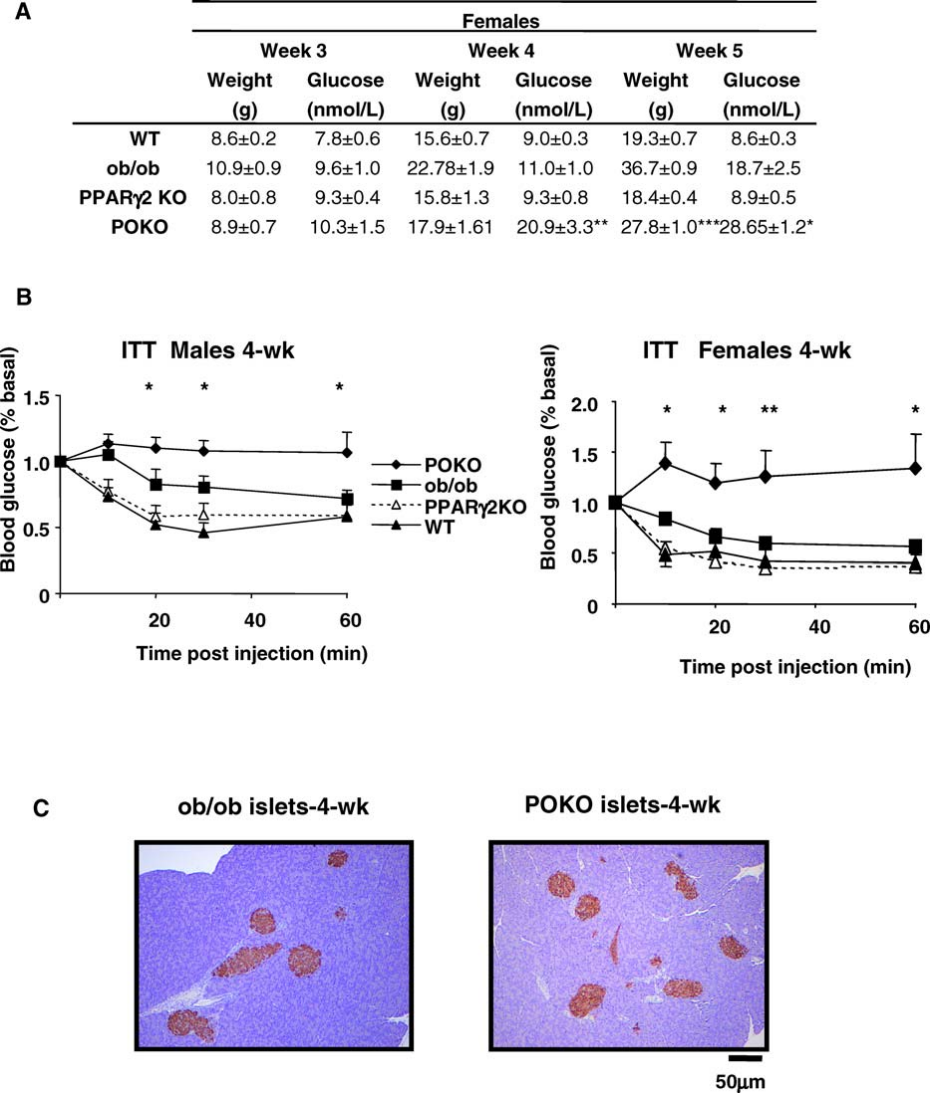
Early Insulin Resistance in POKO Mice Independent of Body Weight (A) Body weight and plasma glucose levels from three, four, and five-week-old female WT, ob/ob, PPARg2 KO, and POKO. *, *p* < 0.05; **, *p* < 0.01; ***, *p* < 0.001 POKO versus ob/ob. (B) Plasma glucose levels during ITT on 4-wk-old male (left) and female (right) mice on chow diet (black triangle, WT; white triangle, PPARg2 KO; black square, ob/ob; black diamond, POKO) (*n* = 7). *, p < 0.05; **, p < 0.01 POKO versus ob/ob. (C) Morphological analysis of H and E-stained sections (10×) in pancreas from 4-wk-old males ob/ob and POKO mice (*n* = 5).

As expected the reduced adipose tissue expandability of the POKO mouse was associated with severe insulin resistance. Surprisingly insulin resistance developed very early in life with elevated insulin levels and blood glucose compared to ob/ob mice (Table 1). We investigated whether peripheral insulin resistance and/or a severe defect in insulin secretion may cause hyperglycaemia in the POKO mouse. No differences in plasma glucose levels were detected three to five days after birth amongst the four genotypes for both genders (unpublished data). At weaning (three weeks of age) total body weight was indistinguishable amongst the four genotypes, and blood glucose levels were similar in males and females (Figure 2A). However, by the age of four weeks, coincident with the change to a chow diet, male and female POKO mice developed severe hyperglycaemia compared to the other genotypes. Insulin plasma levels in the POKO mice at four weeks of age were increased compared to ob/ob mice (Table 1). Insulin resistance in POKO mice was confirmed by an insulin tolerance test (ITT) in four-week-old male and female mice (Figure 2B). Furthermore insulin resistance in adipose tissue was demonstrated by the extremely low levels of glucose transporter4 (GLUT4) protein in POKO adipose tissue when compared with GLUT4 levels in adipose tissue from ob/ob mice (Figure S3). Of note, insulin resistance in the POKO mice was associated with hypertriglyceridaemia as early as four-weeks of age (Table 1).

**Table 1:**
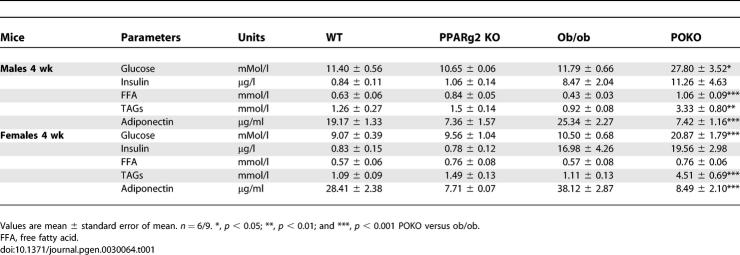
Metabolic Parameters in Fed 4-Wk-Old Male and Female POKO, Ob/Ob, PPARg2 KO, and WT Mice

### Adult POKO Mice are Hyperglycaemic and Have Low Plasma Insulin Levels

Given the early insulin resistance and hyperinsulinaemia in the young POKO mice, we expected to see increased insulin levels in mature POKO mice. At 16 weeks, male POKO mice exhibited severe hyperglycaemia in the fasted and fed states compared to littermate controls. Male POKO mice had inappropriately low levels of insulin (Table 2). A similar, but milder phenotype was also observed in POKO female mice (unpublished data). Of note, adult ob/ob mice compensated for their insulin resistance with increased insulin levels (Table 2). POKO mice also had hypertriglyceridaemia when compared to WT, ob/ob, or PPARg2 KO mice.

**Table 2:**
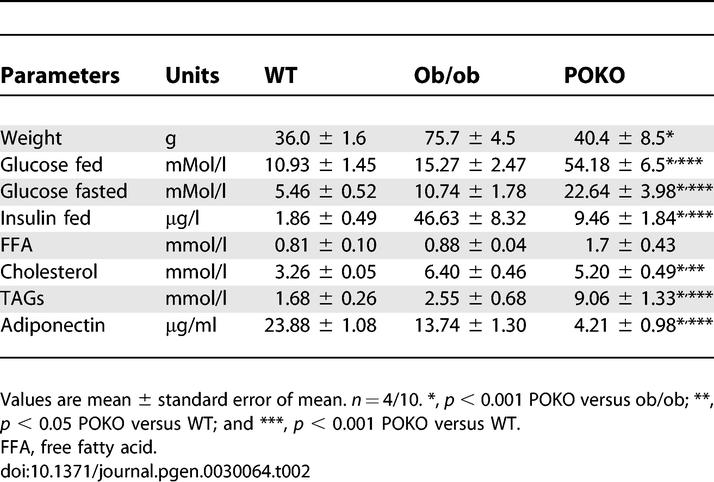
Metabolic Parameters in 16-wk-Old Male POKO, Ob/Ob, and WT Mice

### Impaired Beta-Cell Function in the POKO Mice

**Figure 3:**
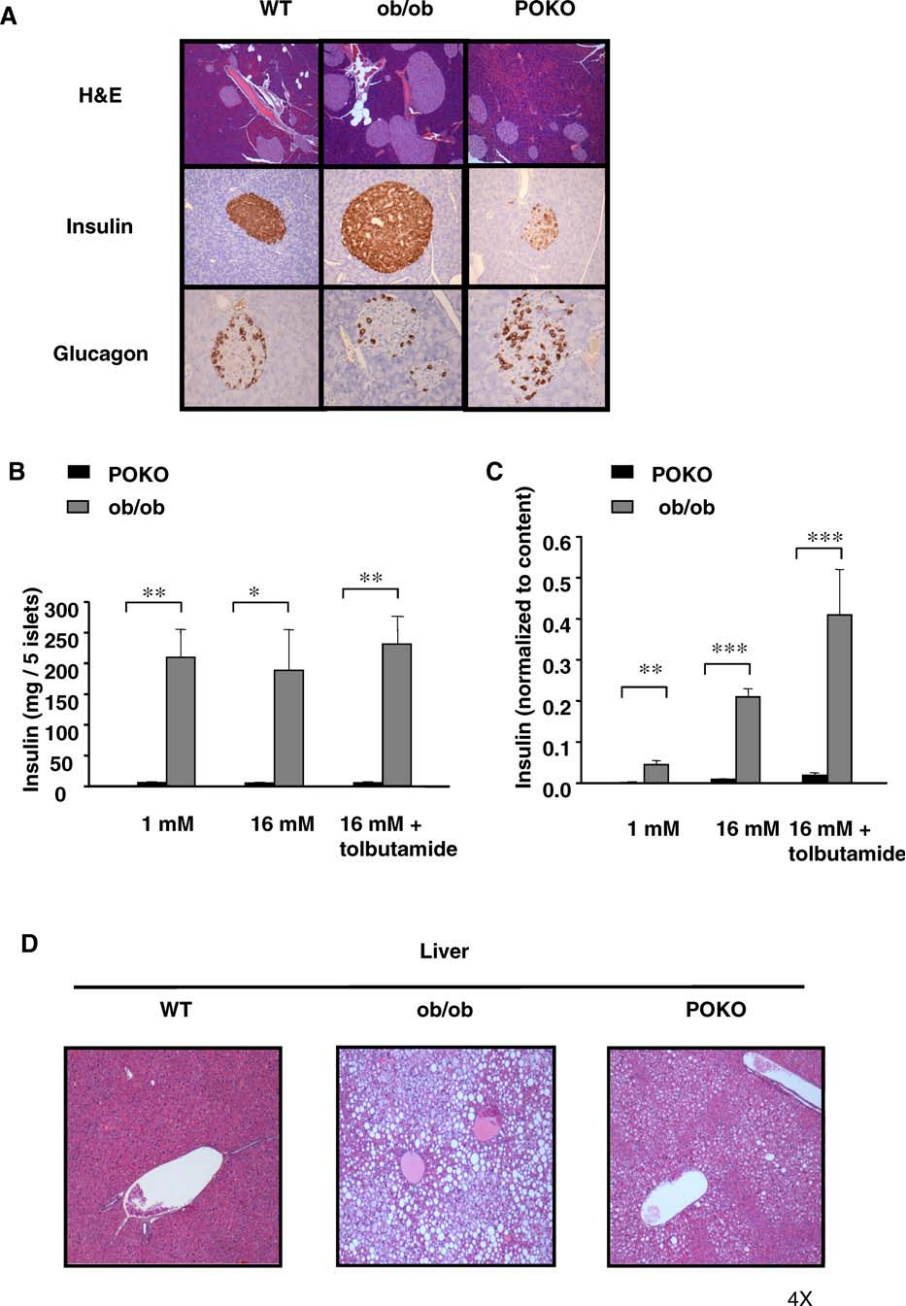
Impaired β-Cell Function and Hepatic Morphological Analyis in the POKO Mice (A) H and E-stained sections (10×) and immunohistochemical (20×) analysis of insulin and glucagon in pancreas from 16-wk-old males WT, ob/ob, and POKO mice (*n* = 5). (B) Insulin content of islets isolated from POKO (black bars), and ob/ob (grey bars) mice. Each data point is the mean of six samples each of five islets.**** (C) Insulin secretion from islets isolated from POKO (black bars) and ob/ob (grey bars) mice in response to glucose (1, 16 mM) or glucose 16 mM + tolbutamide (200 μM). Data were collected from six samples each of five islets from three mice of each genotype. For each sample, insulin release was normalised to insulin content. *, *p* < 0.05; **, *p* < 0.01; ***, *p* < 0.001 POKO versus ob/ob. (D) H and E-stained sections (4×) in liver from 16-wk-old males WT, ob/ob, and POKO mice (*n* = 5).

The inappropriately low insulin levels in the adult POKO mice suggested a defect in β-cells. Insulin resistance in ob/ob mice was compensated for by increasing pancreatic insulin secretion, islet number, and size (Figure 3A). However, despite being more insulin resistant than ob/ob mice, POKO mice did not increase their β-cell mass, resulting in lower plasma insulin levels than the ob/ob controls. Morphometric analysis of pancreatic sections from 16-week-old male mice confirmed that the islet-to-pancreas volume ratios were similar in the POKO, WT, and PPARg2 KO mice (0.023 ± 0.005, 0.013 ± 0.006, and 0.016 ± 0.005, respectively) and markedly increased in ob/ob mice (0.077 ± 0.017, *p* < 0.01 ob/ob versus POKO). Additionally, POKO mice had significantly decreased islet number and size (average area of islets POKO = 18.40 ± 2 mm^2^) compared to ob/ob mice (ob/ob = 61.59 ± 8 mm^2^). Insulin staining demonstrated that islets from POKO mice contained fewer insulin-positive cells than islets from ob/ob mice (Figure 3A). The normal cellular organization of the islet, abundant β-cells (insulin staining) in the centre of the islet and a rim of α-cells at the periphery (glucagon staining), was retained in the insulin resistant ob/ob mice but was disrupted in the islets of POKO mice (Figure 3A). Islets from POKO mice had decreased number of insulin positive β-cells when compared to islets from ob/ob mice and a scattered pattern of α-cells, which are morphological changes associated with islet remodelling in the context of β-cell failure. Gene expression analysis of islets from 16-week-old mice revealed decreased expression of pancreatic duodenal homeobox-1, insulin receptor substrate 2, *Glut2,* and insulin in islets from POKO mice when compared with those from WT or ob/ob (Figure S4).

The changes seen in the β-cells of POKO mice were not the result of an inherent failure of the β-cell to develop properly as indicated by histological studies of neonatal pancreas (day 3 to day 5) (unpublished data) and four-week-old pancreas (Figure 2C), showing no morphological differences in the size, number, or insulin staining of islets from POKO mice when compared to ob/ob controls.

### Impaired Glucose-Stimulated Insulin Secretion in POKO Mouse Islets

We measured glucose-stimulated insulin secretion in 16-week-old female POKO mice and their ob/ob littermates. Islets isolated from POKO mice were 30% smaller than those from ob/ob mice. Moreover, whereas normal islets were pure white with a smooth surface, islets from POKO mice were gray; their surface was irregular and required less time for collagenase digestion (only ten minutes instead of 30 minutes), suggesting that they were also more fragile.

Insulin content in islets from ob/ob mice was more than 30-fold greater than in those from POKO mice (Figure 3B). Insulin secretion from the islets of POKO mice was strikingly impaired compared to those of ob/ob mice, even when expressed relative to insulin content (Figure 3C). This was observed under basal (1 mM glucose) and stimulated (16 mM glucose, 16 mM glucose + tolbutamide) release.

### Decreased Steatosis in POKO Mice Compared to Ob/Ob Mice

As expected, the POKO mice had increased hepatic fat deposition compared to WT and PPARg2 KO mice (Table S1), but surprisingly the POKO mouse had much milder hepatosteatosis than the ob/ob mouse (Figure 3D), suggesting that ectopic expression of the PPARg2 isoform in the liver of ob/ob mice (see below), might contribute to the deposition of TAGs in the liver.

### Ablation of PPARg2 Induces a Lipotoxic Lipid Profile in Adipose Tissue, Pancreatic Islets, Liver, and Skeletal Muscle

To investigate lipotoxicity as a potential pathogenic mechanism we used liquid chromatography/mass spectrometry (LC/MS) [32] to compare a broad spectrum of cellular lipids in the adipose tissue, pancreatic islets, liver, and skeletal muscle between the POKO mouse and controls (Protocol S1).

#### Adipose tissue from POKO mice has decreased TAG but increased DAG, ceramides, and other reactive lipid species associated with insulin resistance.

**Figure 4:**
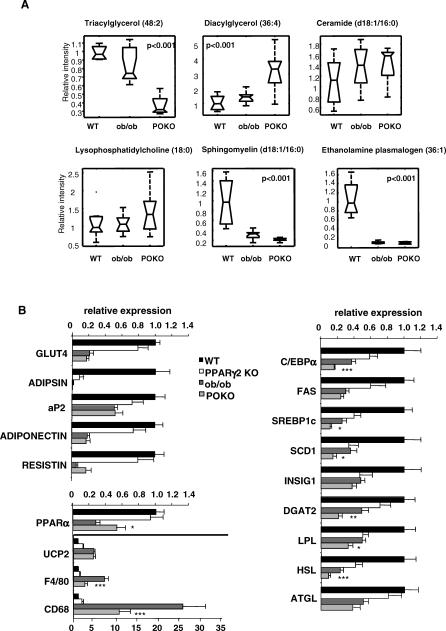
Lipidomic and Gene Expression Analysis of POKO WAT (A) Lipidomic profiling of WAT from 16-wk-old males WT, ob/ob, and POKO mice. (B) Adipose tissue mRNA levels from different genes from 16-wk-old male WT, PPARg2 KO, ob/ob, and POKO mice (*n* = 6–8). *, *p* < 0.05; **, *p* <0.01; ***, *p* <0.001 POKO versus ob/ob.

Lipidomic analysis using LC/MS identified 74 molecular species differentially present in POKO, ob/ob, and WT mice (Protocol S1). POKO adipose tissue had decreased short chain TAGs compared to ob/ob adipose tissue (Protocol S1). Conversely, the concentration of DAGs was increased in the WAT of the POKO mice compared to ob/ob littermates. There was also an increased concentration of reactive lipid species in the WAT of POKO mice compared to that of ob/ob. The WAT of both POKO and ob/ob mice (Protocol S1) had increased levels of two ceramide species (with 16:0 and 24:1 fatty acid chains, respectively) and three proinflammatory lysophosphatidylcholine species [33] compared to WT mice. Partial least squares discriminant analysis indicated these changes in ceramides were greater in the POKO than ob/ob mice (Protocol S1). Sphingomyelin (d18:1/16:0), the precursor of ceramide (d18:1/16:0) and antioxidant ethanolamine plasmalogen (36:1) [34] were markedly decreased in POKO and ob/ob mice (Figure 4A).

#### Decreased TAG and accumulation of reactive lipid species in islets from POKO mice.

**Figure 5:**
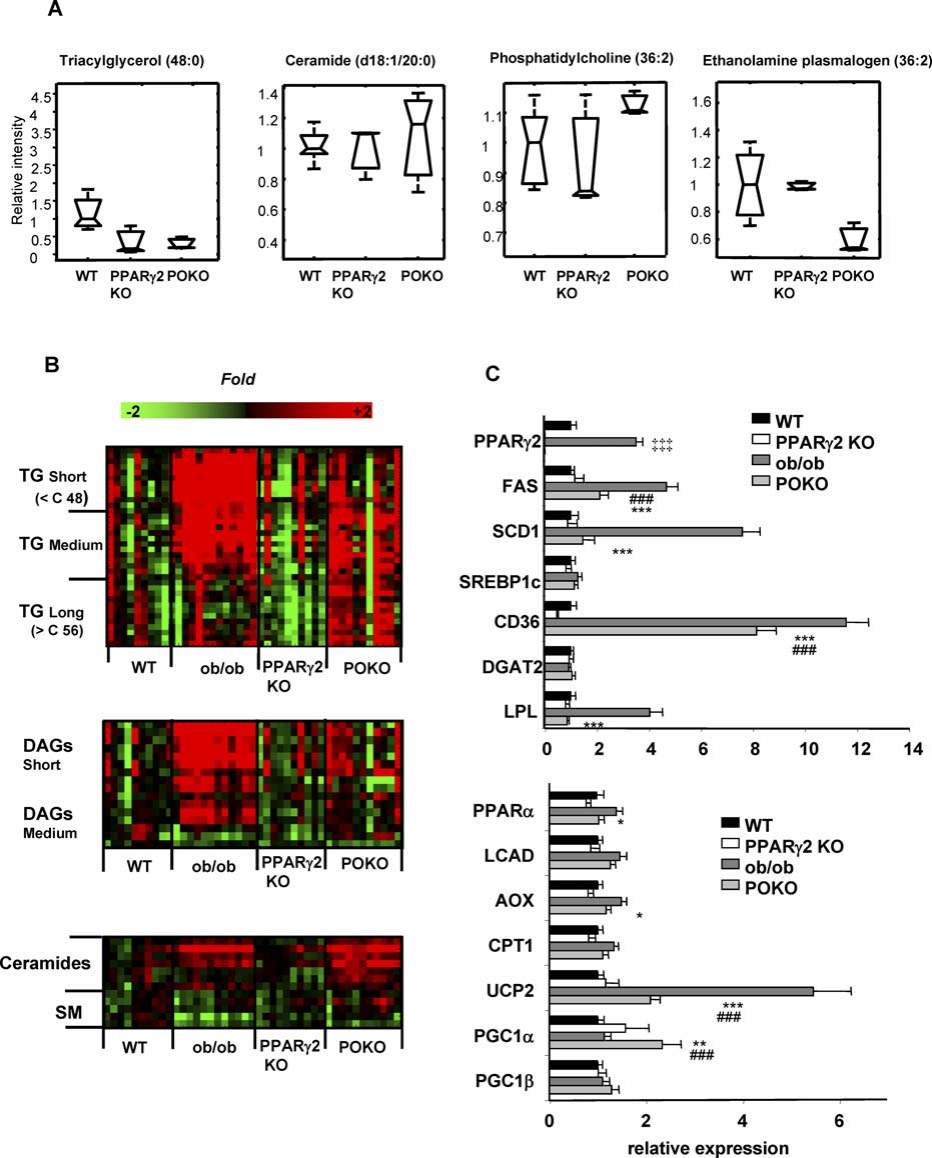
Lipidomic and Gene Expression Analysis in Islets and Liver from POKO Mice Lipidomic profiling of islets (A) and liver (B) from 16-wk-old males WT, PPARg2 KO, ob/ob, and POKO mice. TG, TAGs; DAGs, diacylglycerols; SM, sphingomyelins. (C) Liver gene expression from 16-wk-old male WT, ob/ob, PPARg2 KO, and POKO mice fed chow (*n* = 6–8). *, p < 0.05; **, p, <0.01; ***, p <0.001 POKO versus ob/ob; ###, p < 0.001 POKO versus WT; ‡‡‡, p < 0.001 ob/ob versus WT.

Partial least-squares discriminant analysis of lipidomic profiles of isolated pancreatic islets of 16-week-old mice identified 44 lipid species accumulated at different concentrations in WT, PPARg2 KO, and POKO mice (Protocol S1). Short chain TAGs were decreased in islets from POKO and PPARg2 KO mice when compared to those from WT. This was associated with up-regulation of phosphatidylethanolamine (36:2), down-regulation of ethanolamine plasmalogen (36:2), and preferential accumulation of reactive lipid species, particularly of two ceramides (20:0 and 22:0 fatty acids) in islets from POKO mice (Figure 5A and Protocol S1).

#### Decreased TAG and increased reactive lipid species in liver of POKO mice.

Multivariate analysis of lipidomic profiles (192 lipid species) revealed large changes between the POKO, PPARg2 KO, ob/ob, and WT genotypes (Protocol S1). These included decreased levels of short and medium chain TAGs and DAGs (Figure 5B) in livers from POKO mice compared to those of ob/ob mice. Livers from POKO mice also had decrease levels of phosphatidylcholine lipid species (Protocol S1) utilised during the formation and secretion of very low density lipoproteins [35]. Conversely, POKO livers were enriched in ceramides compared to ob/ob livers, which correlated with the extent of increased levels of lysophosphatidylcholines in POKO and ob/ob mice (Protocol S1).

#### Decreased TAG and accumulation of reactive lipid species in POKO skeletal muscle.

The same lipidomic pattern of decreased TAG and increased reactive lipid species previously observed in adipose tissue, β-cell, and liver was found to a milder degree in the skeletal muscle of POKO mice (Protocol S1). Briefly, when compared to ob/ob skeletal muscle, POKO skeletal muscle showed a decrease in very short-chain fatty acid TAGs and a slight decrease in levels of medium and long chain TAGs (Protocol S1). The skeletal muscle of POKO mice also had increased reactive lipids including ceramide (d18:1/18:0), DAGs, lysophosphatidylcholines, and sphingomyelins (precursors of ceramides) when compared to that of ob/ob mice.

### Transcriptomic Analysis in POKO Mice Correlates with Lipidomic Changes

Given the lipotoxic profiles identified in the POKO mouse, we hypothesised changes in the expression of metabolic genes directly related to PPARg2 ablation and also compensatory changes in genes associated with cellular stress (Table S4).

#### Gene expression analysis in WAT.

Target genes of PPARg such as *Glut4, adipsin, aP2,* and *adiponectin* were decreased to a larger extent in the WAT of five- and 16-week-old POKO mice than in PPARg2 KO mice (Figure S1 and Figure 4B). At five weeks of age, when differences in body fat between female WT, ob/ob, and POKO mice are only starting to become evident, levels of GLUT4, aP2, and adiponectin mRNA levels were similar in WT and ob/ob mice, yet were markedly decreased in POKO mice. As the ob/ob mice aged (16 wk) and became obese and insulin resistant, the expression pattern of these PPARg targets in the WAT of ob/ob mice became similar to that of the POKO mice.

Results from the lipidomic analysis suggested major changes in the expression of genes involved in lipid metabolism (Figure 4B). Expression of stearoyl-coenzyme A desaturase 1 *(Scd1)* and sterol regulatory element-binding protein-1c (SREBP1c) were significantly lower in WAT from POKO mice compared to ob/ob mice. Furthermore, the decrease in TAGs and increased DAGs correlated with decreased expression of DAG acyltransferase 2, a key enzyme catalysing the final step in TAG synthesis, in the WAT of POKO mice compared with WAT from ob/ob mice. Again supporting the lipidomic profile, the expression of hormone-sensitive lipase, a rate-limiting enzyme for hydrolysis of diacylglycerides, was decreased in the WAT of POKO, PPARg2 KO, and ob/ob mice compared with WT mice, with the lowest levels observed in the POKO mice. Adipose triglyceride lipase levels were decreased in ob/ob and POKO compared with WT and PPARg2 KO mice, but without significant differences between ob/ob and POKO mice.

Oxidative stress has recently been suggested as a common mechanism of insulin resistance. Adipose tissue from POKO mice had increased oxidative stress compared to that of ob/ob mice as indicated by decreased gene expression levels of extracellular CuZn-superoxide dismutase, disruption of the glutathione pathway as indicated by decreased levels of gluthatione synthase, and increased levels of peroxidase and several gluthatione transferases (Table S2). We examined macrophage infiltration of adipose tissue as a potential marker of inflammation associated insulin resistance. Expression of CD68 and F4/80, both macrophage markers, was increased in the WAT of both POKO and ob/ob mice compared with WT and PPARg2 KO mice (Figure 4B). However their expression levels were lower in the POKO mice than the ob/ob mice suggesting that macrophage infiltration was not directly related to the exacerbated insulin resistance of the POKO mouse compared to the ob/ob mouse.

#### Gene expression in the POKO liver.

Reduced hepatic steatosis accompanied by altered lipid profiles suggested that lack of hepatic ectopic expression of PPARg2 might be affecting lipid storage and metabolism in the liver of the POKO mice. Expression of genes involved in lipid metabolism in liver (Figure 5C) revealed that, proportional to the accumulation of TAGs in the liver, fatty acid synthase, *Scd1,* and the fatty acid translocase (FAT/CD36) were increased in ob/ob and POKO livers compared to WT mice and were significantly decreased in liver from POKO mice compared with liver from ob/ob mice. Other lipogenic PPARg target genes such as *Lpl* were also decreased in the POKO liver compared to the ob/ob mice. The ob/ob mice also had a compensatory increase in the expression of genes involved in β-oxidation (e.g., *Pparg, Lcad, Aox, Cpt1,* and *Ucp2*). Interestingly expression of these pro-oxidative genes was decreased in the liver of POKO mice when compared to that of ob/ob mice suggesting PPARg2 may contribute to their regulation [36].

Although β-cell failure could account for the severe hyperglycaemia observed in the POKO genotype, hepatic gluconeogenesis function might be affected. We observed a robust up-regulation of PPARg coactivator 1 alpha (PPARGC1a, also known as PGC1a) expression in the POKO liver compared with the WT and ob/ob mice. PPARGC1a is up-regulated in fasting and is thought to induce gluconeogenesis [37]. In parallel with the increase in PPARGC1a, microarray analysis revealed increased mRNA levels of the progluconeogenic genes phosphoenolpyruvate carboxykinase 1 *(Pepck1)* and glucose-6-phosphatase *(G6pc)* in the livers of POKO mice when compared to those of ob/ob mice (Table S2), suggesting hepatic gluconeogenesis may contribute to the hyperglycaemia observed in POKO mice.

#### Gene expression analysis in skeletal muscle of POKO mice.

In 16-week-old POKO-mice skeletal muscle we observed down-regulation of *Srebp1c* and *Ppargc1a* and up-regulation of *Ucp2* expression in skeletal muscle from POKO mice compared to that of WT mice. Similarly, expression of *Lpl* and *Scd1* was down-regulated in the skeletal muscle of POKO mice when compared with that from ob/ob mice (Figure S5; Table S2). Gene set enrichment analysis of microarray data showed decreased expression of oxidative phosphorylation and mitochondrial components including electron transport chain complex components, in skeletal muscle from POKO mice when compared with that from ob/ob mice (Table S3).

## Discussion

The link between obesity, insulin resistance, and diabetes while epidemiologically very clear is still not properly understood at a mechanistic level. An emerging concept is that the absolute amount of fat stored may be less important than the remaining storage capacity of the adipose tissue. Here we show that the PPARg2 isoform may be an important factor controlling obesity-induced comorbidities through two mechanisms: (a) by regulating nutritionally induced adipose tissue expandability and (b) when de novo expressed in nonadipose tissues, by allowing the storage of energy in the form of relatively harmless TAG species.

Previously we described the metabolic phenotype of the adult PPARg2 KO mouse [2], characterised by mild insulin resistance observed only in males. Given the greater adipogenic potency of PPARg2 compared with PPARg1 in vitro, we expected PPARg2 KO mice to have many more severe defects in adipose tissue than we observed, and therefore insulin sensitivity. As PPARg2 is the PPARg isoform regulated in response to nutrition and obesity [17–20], we hypothesised that PPARg2 would only become essential for adipose tissue function in the face of positive energy balance. The metabolic challenge we opted for was PPARg2 ablation in the obese (ob/ob) background (PPARg2^−/−^ Lep^ob^/Lep^ob^, POKO mouse). The POKO mouse had severely decreased body-fat mass due to impaired adipose tissue expandability. Despite eating as much as an ob/ob mouse and expending a similar amount of energy, the POKO mouse was unable to store fat efficiently in its adipose tissue. This mismatch between increased energy availability and lack of adipose tissue expandability lead to a global metabolic failure characterised by severe insulin resistance, β-cell failure, and dyslipidaemia.

The observation of reduced fat mass and increased insulin resistance in the POKO mouse compared to the ob/ob mouse strongly supports two of our hypotheses. First, we hypothesised that PPARg2 is required to recruit new adipocytes in overnutrition, but it is not required to make adipocytes during development. This is reflected by similar expression of aP2, a late marker of adipocyte differentiation, in POKO and ob/ob mice. The absence of small adipocytes was markedly different to other forms of lipodystrophy [38,39]. Additionally, and again in contrast with other lipodystrophic models that have markedly less adipose tissue than WT controls [38–40], the POKO mice had a percentage body fat that was similar (only 4% more) to WT and PPARg2 KO mice, as opposed to ob/ob mice, which had 40% fat as a proportion of body mass. This suggests that the remaining PPARg1 isoform is sufficient to support development of adipose tissue and fat deposition requirements of a lean mouse model. However, under conditions of positive energy balance, adipose tissue expandability mainly relies on the PPARg2 isoform. This idea is also suggested by the studies in heterozygous mice harbouring the murine equivalent of the human mutation *(P465L)* in PPARg on an ob/ob background [41]. These mice were able to accumulate fat and become obese even though showing a body mass 14% lower than ob/ob controls. In humans there is also evidence for a role for PPARg2. We have observed that metabolically healthy, nondiabetic, morbidly obese individuals have elevated levels of PPARg2 in their adipose tissue when compared to lean individuals [19]. Our second hypothesis, that the mismatch between energy availability and adipose tissue expandability is more important than fat mass itself as a predictor of insulin resistance, is also supported by our data. In fact the ob/ob mouse is much more obese than the POKO mouse but is much less insulin resistant. Furthermore, the POKO mice were already more insulin resistant than the ob/ob mice by the age of four weeks, with very low levels of GLUT4 in adipose tissue, before large differences in body weight developed, suggesting that the bioenergetic mismatch rather than the total amount of fat stored is important for the development of insulin resistance.

Although we hypothesised that the POKO mice would become insulin resistant, the degree of hyperglycaemia in these animals was in excess of what we expected. We found that the normal adaptive response of β-cells to insulin resistance did not occur in the POKO mice as indicated by the pathological changes observed by histology and the lack of β-cell hypertrophy. Although it has been shown that genetic background can affect the ability of ob/ob mice to undergo β-cell hypertrophy [42,43], we found that the ob/ob controls on our mixed 129Sv × C57BL/6J background underwent adaptive β-cell hyperplasia and hypertrophy, suggesting that the lack of PPARg2 was responsible for the failure of the POKO β-cells to adapt to insulin resistance. Interestingly the mass of pancreatic islets in POKO mice remained similar to the noninsulin resistant WT and PPARg2 KO mice. Furthermore, these defects in POKO β-cells did not appear to be the result of a developmental defect, as new born and four-week-old mice had morphologically normal islets.

The severe β-cell phenotype of the POKO mouse contrasts with the absence of hyperglycaemia observed in the pancreatic β-cell specific PPARg KO mouse [30]. However it should be kept in mind that in the β-cell specific PPARg KO mouse, the expression of PPARg and the lipid storage capacity of other tissues, most importantly adipose tissue, were not affected, and that insulin sensitivity was only mildly affected by high fat feeding in these mice when compared to the severe insulin resistance observed in POKO mice. Therefore the challenge to the pancreatic β-cells in this model was milder than in POKO mice. This is a clear example of how tissue-specific genetic manipulations are not always the best approach to understand the physiology of an organ in the context of the global energy homeostasis. The potential importance of the de novo expression of PPARg2 isoform in β-cells is also supported by the observation that humans harbouring the Pro12Ala mutation in PPARg2, a mutation that is located in the g2 isoform and makes PPARg2 less active, has only been associated with insulin deficiency and disease severity in obese individuals with type 2 diabetes [44].

The liver of the POKO mouse also displayed an unusual phenotype. We expected the POKO mice to have worse hepatosteatosis with increased triglyceride deposition in liver compared to ob/ob mice, because the POKO mice could not store fat in adipose tissue. However POKO mice had less hepatosteatosis than ob/ob mice suggesting that the PPARg2 isoform may directly contribute to facilitate triglyceride deposition in the liver.

A common mechanistic link for the phenotypes observed in the POKO liver and β-cell was not immediately obvious. To try to determine the role of PPARg2 in these locations we performed lipidomic and gene expression analyses of the adipose tissue, pancreatic islet, liver, and skeletal muscle of the POKO mouse. The lipid pattern of adipose tissue from POKO mice was characterised by decreased TAGs and increased DAGs in parallel with decreased gene expression of DGAT2, hormone-sensitive lipase, and adipose triglyceride lipase. This decrease in TAGs in the POKO adipose tissue was associated with increased levels of reactive lipid species and a gene expression profile suggestive of increased oxidative stress [45–49]. Although it has been described that oxidative stress and insulin resistance may be related to infiltration of adipose tissue by macrophages, resulting in a chronic state of inflammation [50–52], we did not observe increased macrophage infiltration in the adipose tissue of POKO mice compared to that of ob/ob mice.

Lipidomic analysis of POKO derived islets also showed decreased levels of triacyl and DAGs and increased levels of ceramides, suggesting that PPARg2 may contribute to increasing the lipid-buffering capacity of β-cells by promoting formation of TAGs and thus preventing lipotoxic insults. Liver and skeletal muscle lipidomics also showed reduced TAG and increased formation of reactive lipid species such as ceramides and lysophosphatidylcholines in POKO mice compared to ob/ob mice. This lipid profile was associated with impaired expression of pathways controlling de novo lipogenesis, transport of fatty acids, and beta oxidation in the POKO mice compared with the ob/ob mice. Of interest, *Ppargc1a* and other gluconeogenic genes were induced in the liver of POKO mice compared to that of ob/ob mice, suggesting a potential mechanism contributing to marked hyperglycaemia in POKO mice [53,54].

**Figure 6:**
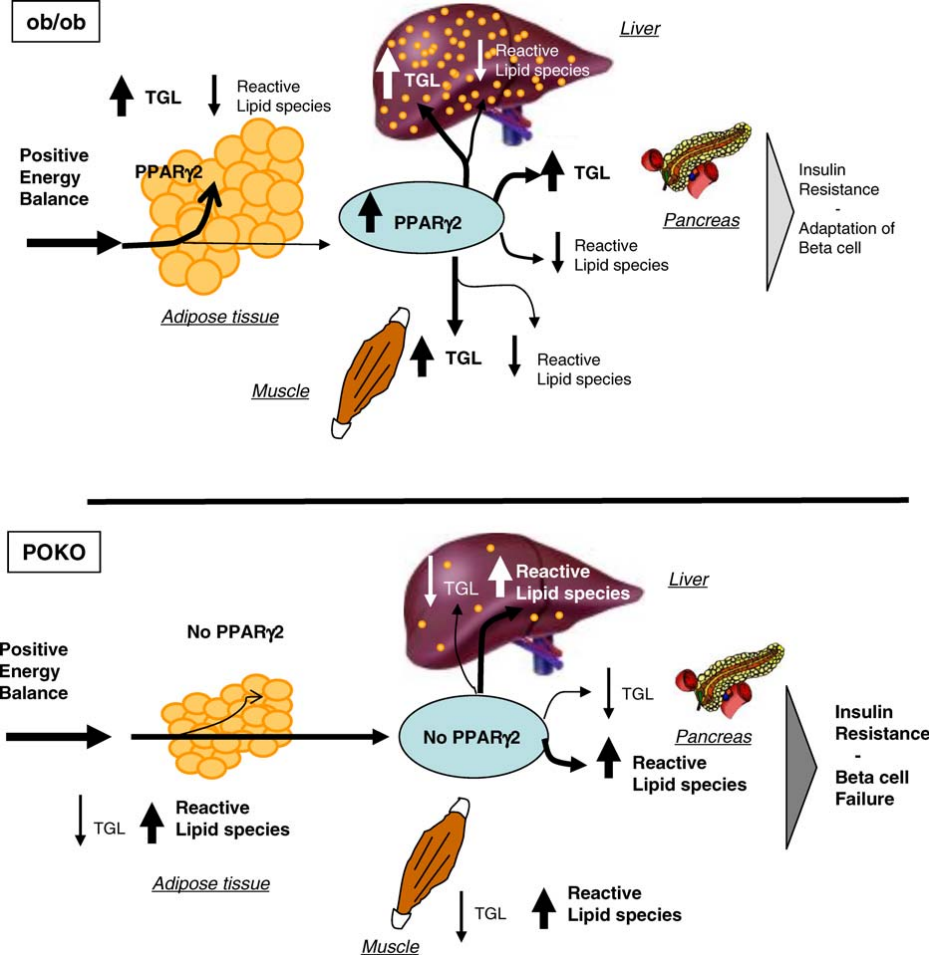
Storage of Lipids—Antilipotoxic Role of PPARg2 Antilipotoxic role of PPARg2 mediated by (a) expansion of adipose tissue and facilitation of triglyceride deposition and (b) facilitating deposition of fat in liver, skeletal muscle, and pancreas in the form of TAG. Ob/Ob mice can induce PPARg2 expression in liver, muscle, and β-cell, facilitating deposition of excess of energy in these organs in the form of TAG. Absence of inducibility of PPARg2 in POKO mouse liver, muscle, and β-cells results in increased deposition of reactive lipid species and decreased TAG, leading to marked insulin resistance and β-cell failure.

Overall, our lipidomic studies identify a remarkably similar pattern of changes in lipid species in the four tissues studied. The reduced adipose tissue mass and hepatosteatosis in the POKO mouse compared to the ob/ob mouse is explained by reduced levels of mature TAG in the POKO mouse. Similarly, ablation of PPARg2 resulted in accumulation of reactive lipid species implicated in causing insulin resistance, not only in adipose tissue, but also in other organs involved in whole-organism glucose metabolism. These results indicate that expression of PPARg2 in the pancreas, liver, and muscle of the ob/ob mouse may be performing a protective role, by increasing the capacity of these organs to buffer toxic lipid species by allowing accumulation of relatively harmless TAGs. The importance of this peripheral antilipotoxic role of PPARg2 becomes more evident if we consider that POKO and ob/ob mice are under the same degree of positive energy balance as determined by similar food intake, locomotor activity, and energy expenditure, that both models lack leptin, and that the only difference between ob/ob and POKO mice is the presence or absence of PPARg2. Given the decreased adipose tissue expandability of the POKO mice compared to ob/ob, it was anticipated that, as in the liver, muscle, or β-cells of lipodistrophic mice, the POKO mouse would accumulate more fat than the ob/ob. However, our results clearly indicate that mice lacking PPARg2, despite massive nutrient availability, are unable to deposit TAG in peripheral tissues and instead accumulate reactive lipid species in these organs. Therefore the pathologies of the liver and β-cell observed in the POKO mouse may be a result of a common lipotoxic insult facilitated by the absence of PPARg2 (Figure 6).

In summary, in this study we provide new insights into the physiological relevance of the PPARg2 isoform and identify adipose tissue expandability as an important determinant of metabolic complications. Ablation of PPARg2 decreases adipose tissue expandability, but its pathophysiological effects only become relevant in the context of a mismatch between energy availability and adipose tissue expansion. We show that PPARg2 also plays protective role when expressed de novo in peripheral organs by increasing their capacity to buffer toxic lipids. Ablation of PPARg2 under conditions of positive energy balance determined by absence of leptin produced early development of severe insulin resistance, β-cell failure, diabetes, and hyperlipidaemia. Extrapolation of this model to humans may suggest that normal to overweight individuals with positive energy balance and inappropriately severe manifestations of the MS may have a defect in PPARg2 and/or alternative mechanisms that control adipose tissue expandability.

## Materials and Methods

### Generation of mice homozygous for PPARg2 KO and leptin deficiency (ob/ob).

Mice heterozygous for a disruption in exon B1 of PPARg2 on a 129Sv background (PPARg2^+/−^) [2] were crossed with heterozygous ob/ob (Lep^ob^/Lep^+^) mice on a C57Bl/6 background to obtain mice heterozygous for both the PPARg2 ablation and the leptin point mutation (PPARg2^+/−^ Lep^ob^/Lep^+^). These mice were crossed to obtain the four experimental genotypes: WT (PPARg2^+/+^ Lep^+^/Lep^+^), PPARg2 KO (PPARg2^−/−^ Lep ^+^/Lep^+^), ob/ob (PPARg2^+/+^ Lep^ob^/Lep^ob^), and POKO (PPARg2^−/−^ Lep^ob^/Lep^ob^). Genotyping for deletion of PPARg2 and the point mutation in the ob gene was performed by PCR using standard protocols [2,55].

### Animal care.

Animals were housed at a density of four animals per cage in a temperature-controlled room (20–22 °C) with 12-h light/dark cycles. Food and water were available ad libitum unless noted. All animal protocols used in this study were approved by the UK Home Office and the University of Cambridge.

### Blood and urine biochemistry, food intake, and body composition analysis.

Mice of the four experimental genotypes were placed at weaning (three weeks of age) on a normal chow diet (10% of calories derived from fat; D12450B, Research Diets, http://www.researchdiets.com). Enzymatic assay kits were used for determination of plasma FFAs (Roche, http://www.roche.com) and TAGs (Sigma-Aldrich, http://www.sigmaaldrich.com). Elisa kits were used for measurements of leptin (R & D Systems, http://www.rndsystems.com), insulin (DRG Diagnostics International Limited, http://www.drg-international.com), and adiponectin (B-Bridge International, http://www.b-bridge.com) according to manufacturers' instructions. Dual-energy X-ray absorptiometry (DEXA, Lunar Corporation, http://www.lunarcorp.com) was used to measure body composition; glucose in blood and in urine and food intake were monitored in the four experimental genotypes as previously shown [2].

### Oxygen consumption, water intake, and locomotor activity.

Oxygen was measured using an eight-chamber open-circuit oxygen-monitoring system attached to and sampled from the chambers of a Comprehensive Laboratory Animal Monitoring System (CLAMS; Columbus Instruments, http://www.colinst.com). Water consumed was also measured using CLAMS. Mice were housed individually in specially built Plexiglass cages maintained at 22 °C under an alternating 12:12-h light-dark cycle (light period 08:00–20:00). Sample air was sequentially passed through oxygen (O_2_) and carbon dioxide (CO_2_) sensors (Columbus Instruments) for determination of O_2_ and CO_2_ content. Mice were acclimatized to monitoring cages for 72 h before data collection. Mice were weighed before each trial. Ambulatory activity of individually housed mice was evaluated using an eight-cage rack OPTO-M3 Sensor system (Columbus Instruments). Cumulative ambulatory activity counts were recorded every 5 min throughout the light and dark cycles.

### Calculations of energy lost in urine.

Energy lost in urine was calculated accordingly as previously shown before [56] using the following calculations:

Energy lost in urine kJ/day = (glucose in urine [mMol/l]/1,000) × molecular weight glucose × (water intake [ml/day]/1,000) × E density_carb_; E density_carb_ = energy density related to oxidations within the body for carbohydrates as glucose = 15.76 kJ/g.

### RNA preparation and real-time quantitative RT-PCR.

Total RNA was isolated from islets and tissues samples according to the manufacturer's instructions (RNAeasy kit, Qiagen, http://www.qiagen.com) and STAT60 (Tel-Test, http://www.isotexdiagnostics.com/tel-test.html). Real-time quantitative PCR was performed using a TaqMan 7900 (Applied Biosystems, http://www.appliedbiosystems.com) according to standard protocols.

### Western blot analyses.

The tissue samples (40 μg) were subjected to SDS-PAGE on 8% polyacrylamide gels. Proteins were then electrophoretically transferred to polyvinylidene difluoride filters. After transferring, the filters were blocked with 5% nonfat dry milk in TBS-Tween 20 followed by incubation with primary GLUT4 and extracellular signal-regulated kinase 1/2 (ERK1/2) antibodies (Promega, http://www.promega.com) overnight. The bands were quantified by scanning densitometry.

### Light microscopy and immunohistochemcal analysis.

Tissue samples for morphological and immunohistochemcal analysis were prepared according to published protocols [2]. Morphometric analyses of adipose tissue and pancreas sections were acquired using a digital camera and microscope (Olympus BX41, http://www.olympus.com), and cell areas were measured using AnalySIS software (Soft Imaging System, http://www.soft-imaging.net). For adipose tissue, two fields from each section were analysed to obtain the mean cell-area per animal (*n* = 5 per genotype). The Computer Assisted Stereology Toolbox (CAST) 2.0 system from Olympus was used to perform all measurements in the pancreas according to published protocols [57].

### Isolation and culture of pancreatic islets.

The pancreas was injected via the bile duct with cold Hank's solution containing 0.4% (w/v) liberase (Roche). The pancreas was removed, digested for 15–30 min, and islets collected by handpicking. Isolated islets were cultured overnight in h-cell medium (SBMI 06, hcell technology, http://www.hcell.com) at 37 °C in 5% CO_2_ in air. Islets were used the day after isolation for insulin secretion studies or RNA extraction.

### Insulin secretion studies.

Insulin secretion from isolated islets (five islets/well) was measured during 1-hr static incubations in Krebs—Ringer Buffer containing either 1 mM glucose, 16.7 mM glucose, or 16.7 mM glucose plus 200 μM tolbutamide in DMSO. The supernatants were assayed for insulin. Insulin content was extracted using 95:5 ethanol/acetic acid. Insulin was measured using a Mouse Insulin ELISA kit (Mercodia, http://www.mercodia.com). Islets were isolated from three mice of each genotype for these experiments. Thus, the data are the mean of three separate experiments, in which data were collected for each test solution from six samples each of five islets. For each sample, insulin release was normalised to insulin content.

### ITT.

ITTs on four-week-old mice were performed as previously published [58].

### Lipid profiling.

For WAT and muscle, the tissue sample (50 mg) was homogenized with 0.15 M sodium chloride (300 μl), and the lipids were extracted with 2 ml of chloroform: methanol (2:1) and used for LC/MS as previously described [2].

For liver and islets, an aliquot (20 μl for liver or 10 μl for islets) of an internal standard mixture (11 reference compounds at concentration level 8–10 μg/ml), 50 μl of 0.15 M sodium chloride (for liver), and chloroform:methanol (2:1) (200 μl for liver or 90 μl for islets) was added to the tissue sample (20–30 mg). The sample was homogenized, vortexed (2 min for liver or 15 s for islets), let to stand (1 h for liver, 20 min for islets), and centrifuged at 10,000 RPM for 3 min. From the separated lower phase, an aliquot was mixed with 10 μl of a labelled standard mixture (three stable isotope-labelled reference compounds at concentration level 9–11 μg/ml), and 0.5–1.0 μl injection was used for LC/MS analysis.

Total lipid extracts were analysed on a Waters Q-Tof Premier mass spectrometer (http://www.waters.com) combined with an Acquity Ultra Performance LC (UPLC). The column, which was kept at 50 °C, was an Acquity UPLC BEH C18 10 × 50 mm with 1.7 μm particles. The binary solvent system (flow rate 0.200 ml/min) included A, water (1% 1 M NH_4_Ac, 0.1% HCOOH), and B, LC/MS grade (Rathburn, http://www.rathburn.co.uk) acetonitrile/isopropanol (5:2, 1% 1 M NH_4_Ac, 0.1% HCOOH). The gradient started from 65% A/35% B, reached 100% B in 6 min, and remained there for the next 7 min. The total run time per sample, including a 5 min re-equilibration step, was 18 min. The temperature of the sample organizer was set at 10 °C.

Mass spectrometry was carried out on Q-Tof Premier (Waters) run in ESI+ mode. The data were collected over the mass range of m/z 300–1,200 with scan duration of 0.2 s. The source temperature was set at 120 °C, and nitrogen was used as desolvation gas (800 l/h) at 250 °C. The voltages of the sampling cone and capillary were 39 V and 3.2 kV, respectively. Reserpine (50 μg/l) was used as the lock spray reference compound (5 μl/min; 10 s-scan frequency).

Data processing was performed using the MZmine software [59]. Identification was performed based on an internal reference database of lipid species, or alternatively utilizing the tandem mass spectrometry. The statistical analyses were performed using Matlab (Mathworks, http://www.mathworks.com) and the Matlab library PLS Toolbox (Eigenvector Research, http://www.eigenvector.com).

Tandem mass spectrometry was used for the identification of selected lipid species. MS/MS runs were performed by using ESI+ mode, collision energy ramp from 15–30 V, and mass range starting from m/z 150. The other conditions were as shown in the Protocol S1.

### Statistics.

Results were expressed as mean ± standard error of mean. Statistical analysis was performed using a two-tailed unpaired t-test between appropriate pairs of groups, and significance declared if *p-*values were less than 0.05.

## Supporting Information

Figure S1 Adipose Tissue and Liver Gene Expression (39 KB PPT)

Figure S2 Water Consumed and Locomotor Activity (38 KB PPT)

Figure S3 GLUT4 protein expression in WAT (65 KB PPT)

Figure S4 Gene Expression in Islets (25 KB PPT)

Figure S5 Gene Expression in Muscle (32 KB PPT)

Protocol S1 POKO Mouse Model Lipidomics Dataset (208 KB PDF)

Table S1 Tissue Weights of 16-Wk-Old Male POKO, Ob/Ob, PPARg2 KO, and WT mice (29 KB PPT)

Table S2 Microarray Data (105 KB DOC)

Table S3 Pathway Analysis from Microarray Data (112 KB XLS)

Table S4 Accession Numbers GenBank (http://www.ncbi.nlm.nih.gov/Genbank) accession numbers for the genes and gene products discussed in this paper.
(58 KB DOC)

## Abbreviations

DAG: diacylglycerol
GLUT: glucose transporter
H and E: haematoxylin and eosin
ITT: insulin tolerance test
LC/MS: liquid chromatography/mass spectrometry
MS: Metabolic Syndrome
PPARg: peroxisome proliferator activated receptor gamma
PPARg2: peroxisome proliferator activated receptor gamma 2
PPARGC1a: PPARg coactivator 1 alpha
*Scd1*: stearoyl-coenzyme A desaturase 1
TAG: triacylglycerol
WAT: white adipose tissue
WT: wild type
