# Supplementary material for: PPAR gamma 2 Prevents Lipotoxicity by Controlling Adipose Tissue Expandability and Peripheral Lipid Metabolism
Source: PLoS Genet. 2007 Apr 27;3(4):e64. doi: 10.1371/journal.pgen.0030064 (PMC1857730; doi:10.1371/journal.pgen.0030064)
Supplement: Figure S1 — (39 KB PPT) [file pgen.0030064.sg001.ppt]

## Slide 1
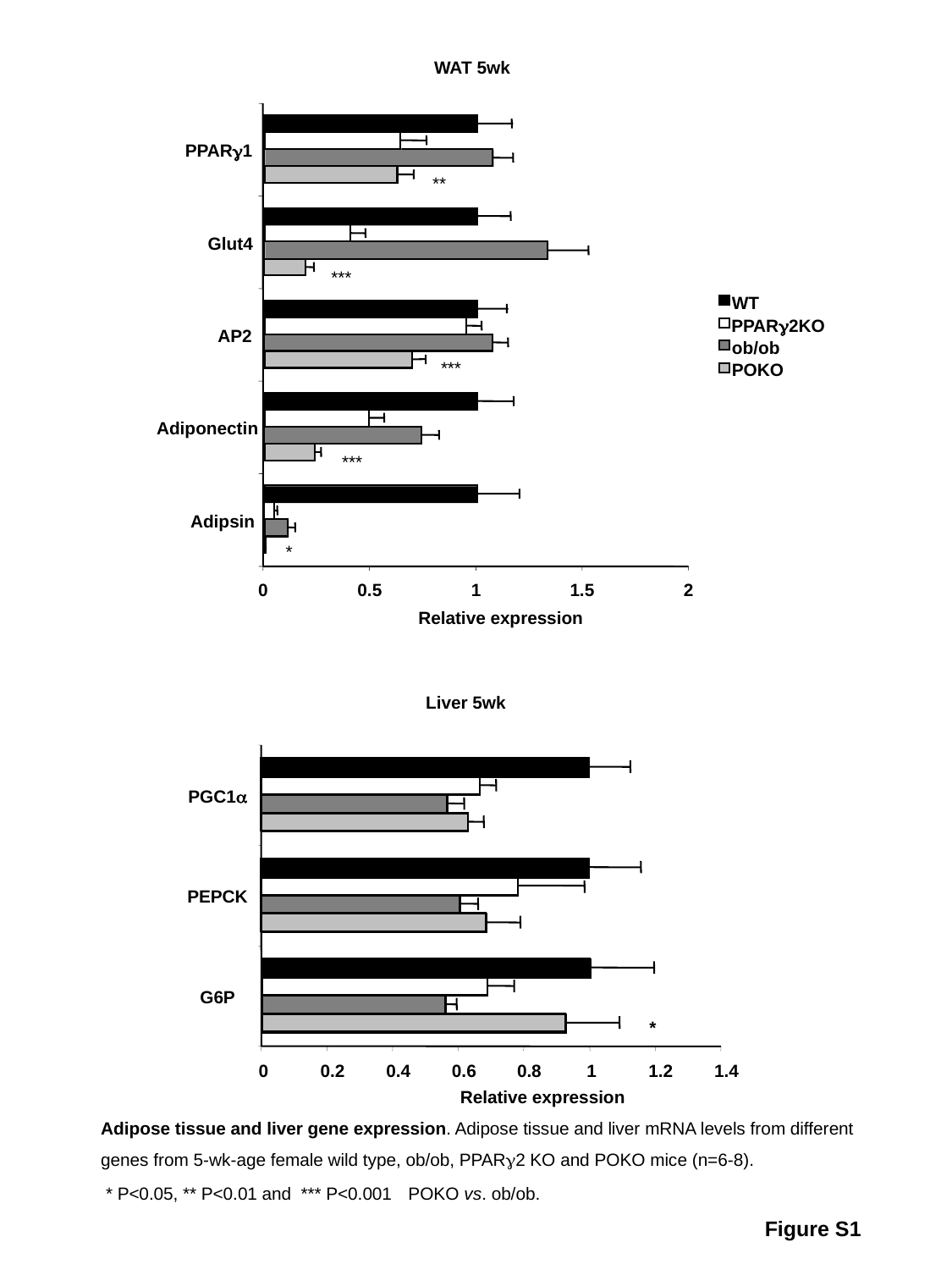

WAT 5wk
PPARγ1
**
Glut4
***
WT
PPARγ2KO
AP2
ob/ob
***
POKO
Adiponectin
***
Adipsin
*
0
0.5
1
1.5
2
Relative expression
Liver 5wk
PGC1α
PEPCK
G6P
*
0
0.2
0.4
0.6
0.8
1
1.2
1.4
Relative expression
Adipose tissue and liver gene expression. Adipose tissue and liver mRNA levels from different
genes from 5-wk-age female wild type, ob/ob, PPARγ2 KO and POKO mice (n=6-8).
 * P<0.05, ** P<0.01 and *** P<0.001 POKO vs. ob/ob.
Figure S1
