# Supplementary material for: PPAR gamma 2 Prevents Lipotoxicity by Controlling Adipose Tissue Expandability and Peripheral Lipid Metabolism
Source: PLoS Genet. 2007 Apr 27;3(4):e64. doi: 10.1371/journal.pgen.0030064 (PMC1857730; doi:10.1371/journal.pgen.0030064)
Supplement: Figure S2 — (38 KB PPT) [file pgen.0030064.sg002.ppt]

## Slide 1
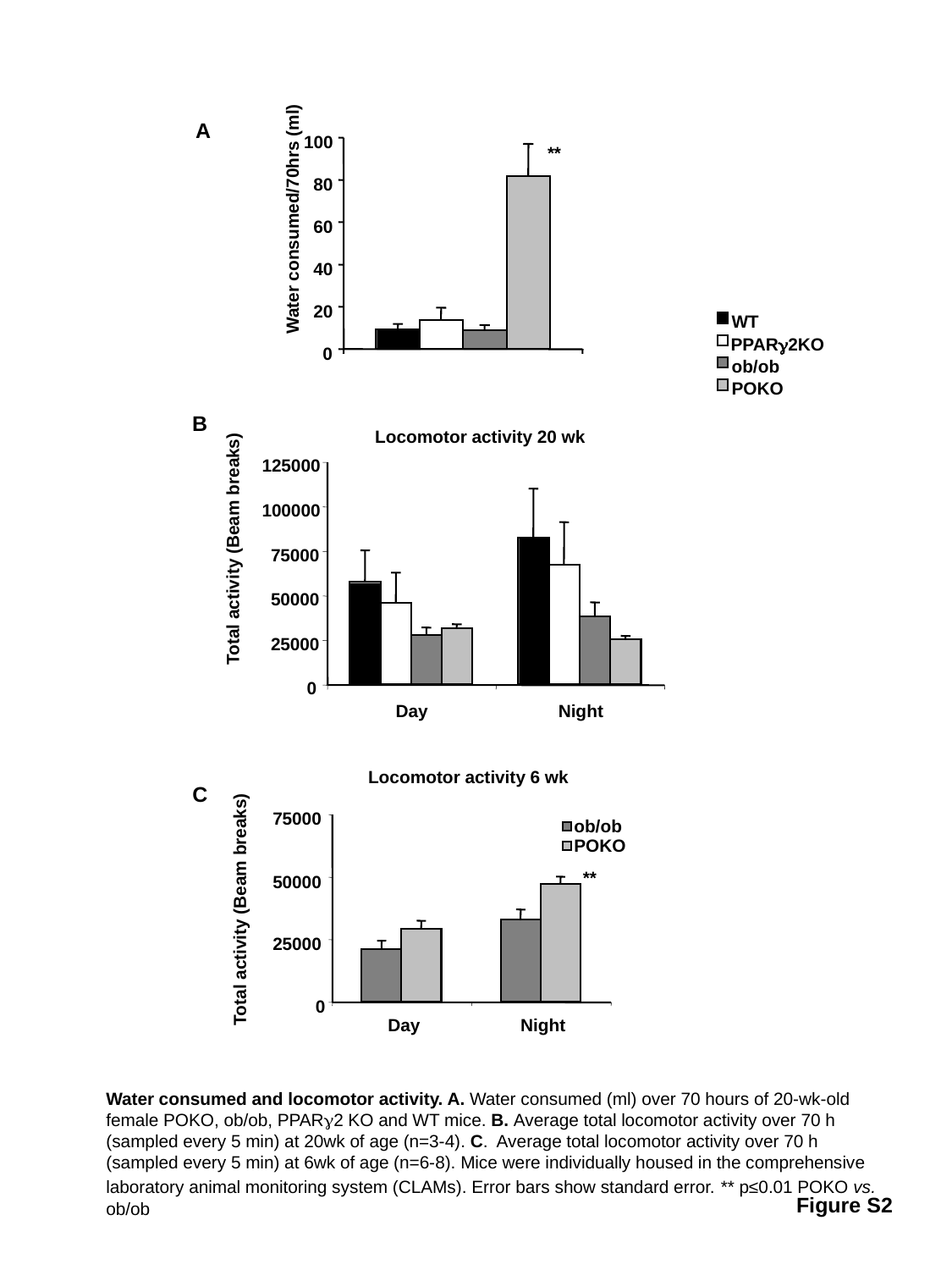

A
100
**
80
Water consumed/70hrs (ml)
60
40
20
WT
PPARγ2KO
0
ob/ob
POKO
 B
125000
100000
Total activity (Beam breaks)
75000
50000
25000
0
Day
Night
Locomotor activity 20 wk
Locomotor activity 6 wk
C
75000
ob/ob
POKO
**
50000
Total activity (Beam breaks)
25000
0
 Day
 Night
Water consumed and locomotor activity. A. Water consumed (ml) over 70 hours of 20-wk-old female POKO, ob/ob, PPARγ2 KO and WT mice. B. Average total locomotor activity over 70 h (sampled every 5 min) at 20wk of age (n=3-4). C. Average total locomotor activity over 70 h (sampled every 5 min) at 6wk of age (n=6-8). Mice were individually housed in the comprehensive laboratory animal monitoring system (CLAMs). Error bars show standard error. ** p≤0.01 POKO vs. ob/ob
Figure S2
