# Supplementary material for: PPAR gamma 2 Prevents Lipotoxicity by Controlling Adipose Tissue Expandability and Peripheral Lipid Metabolism
Source: PLoS Genet. 2007 Apr 27;3(4):e64. doi: 10.1371/journal.pgen.0030064 (PMC1857730; doi:10.1371/journal.pgen.0030064)
Supplement: Figure S3 — (65 KB PPT) [file pgen.0030064.sg003.ppt]

## Slide 1
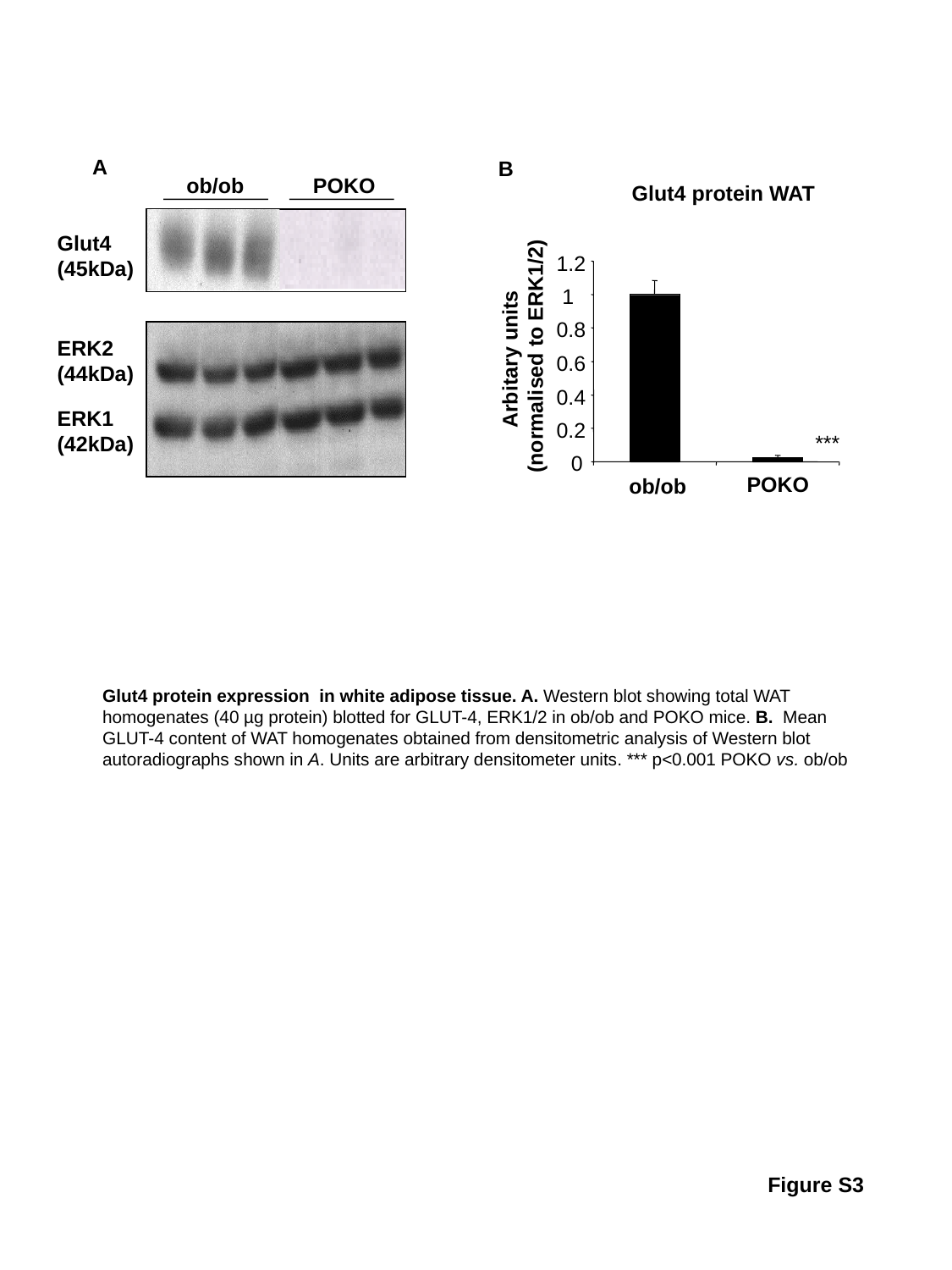

A
B
ob/ob
POKO
Glut4 protein WAT
Glut4
(45kDa)
1.2
1
0.8
ERK2
(44kDa)
Arbitary units
(normalised to ERK1/2)
0.6
0.4
ERK1
(42kDa)
0.2
***
0
POKO
ob/ob
Glut4 protein expression in white adipose tissue. A. Western blot showing total WAT homogenates (40 µg protein) blotted for GLUT-4, ERK1/2 in ob/ob and POKO mice. B. Mean GLUT-4 content of WAT homogenates obtained from densitometric analysis of Western blot autoradiographs shown in A. Units are arbitrary densitometer units. *** p<0.001 POKO vs. ob/ob
Figure S3
