# Supplementary material for: PPAR gamma 2 Prevents Lipotoxicity by Controlling Adipose Tissue Expandability and Peripheral Lipid Metabolism
Source: PLoS Genet. 2007 Apr 27;3(4):e64. doi: 10.1371/journal.pgen.0030064 (PMC1857730; doi:10.1371/journal.pgen.0030064)
Supplement: Figure S4 — (25 KB PPT) [file pgen.0030064.sg004.ppt]

## Slide 1
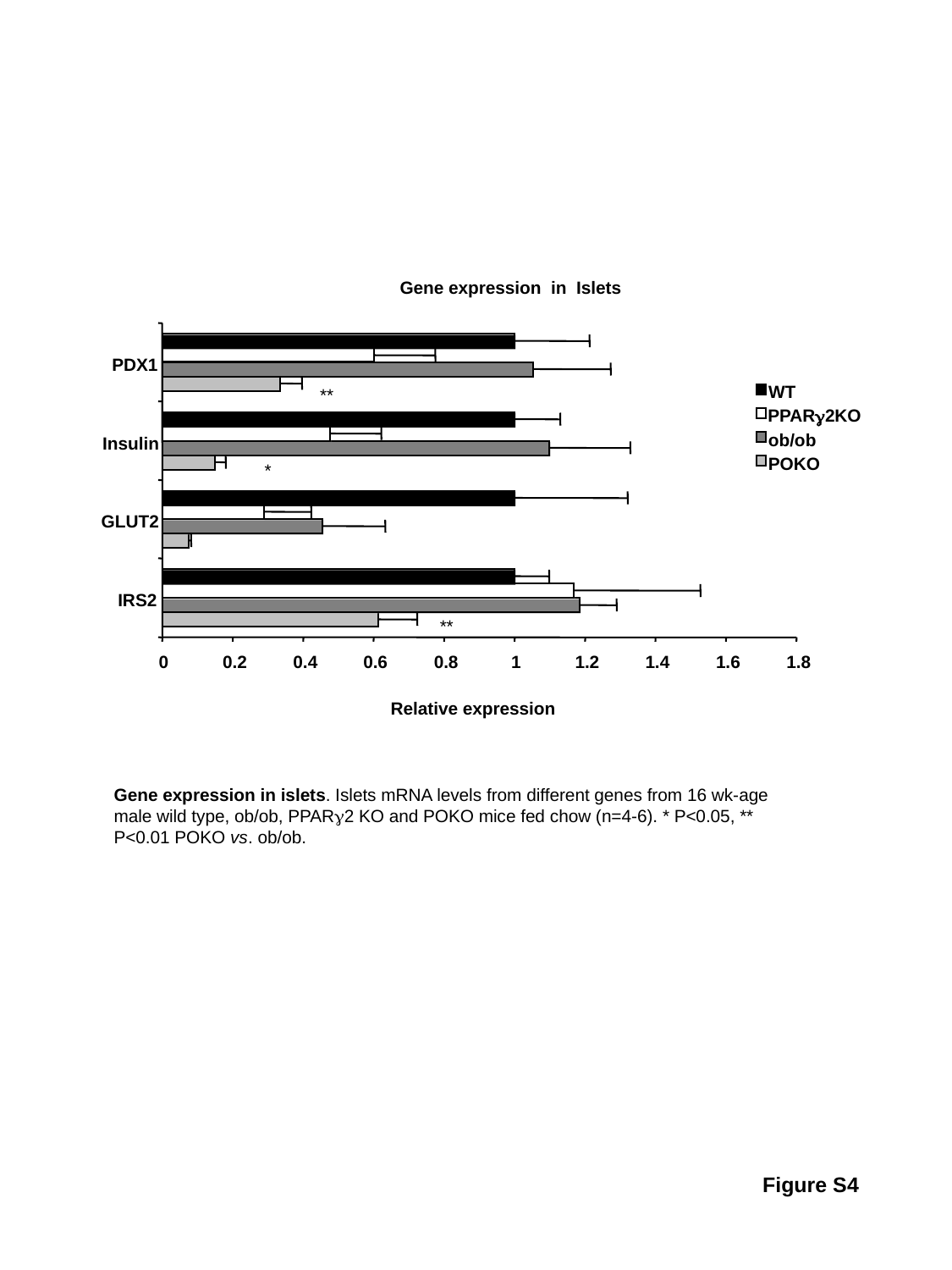

Gene expression in Islets
PDX1
 **
WT
PPARγ2KO
ob/ob
POKO
Insulin
 *
GLUT2
IRS2
 **
0
0.2
0.4
0.6
0.8
1
1.2
1.4
1.6
1.8
Relative expression
Gene expression in islets. Islets mRNA levels from different genes from 16 wk-age male wild type, ob/ob, PPARγ2 KO and POKO mice fed chow (n=4-6). * P<0.05, ** P<0.01 POKO vs. ob/ob.
Figure S4
