# Supplementary material for: PPAR gamma 2 Prevents Lipotoxicity by Controlling Adipose Tissue Expandability and Peripheral Lipid Metabolism
Source: PLoS Genet. 2007 Apr 27;3(4):e64. doi: 10.1371/journal.pgen.0030064 (PMC1857730; doi:10.1371/journal.pgen.0030064)
Supplement: Figure S5 — (32 KB PPT) [file pgen.0030064.sg005.ppt]

## Slide 1
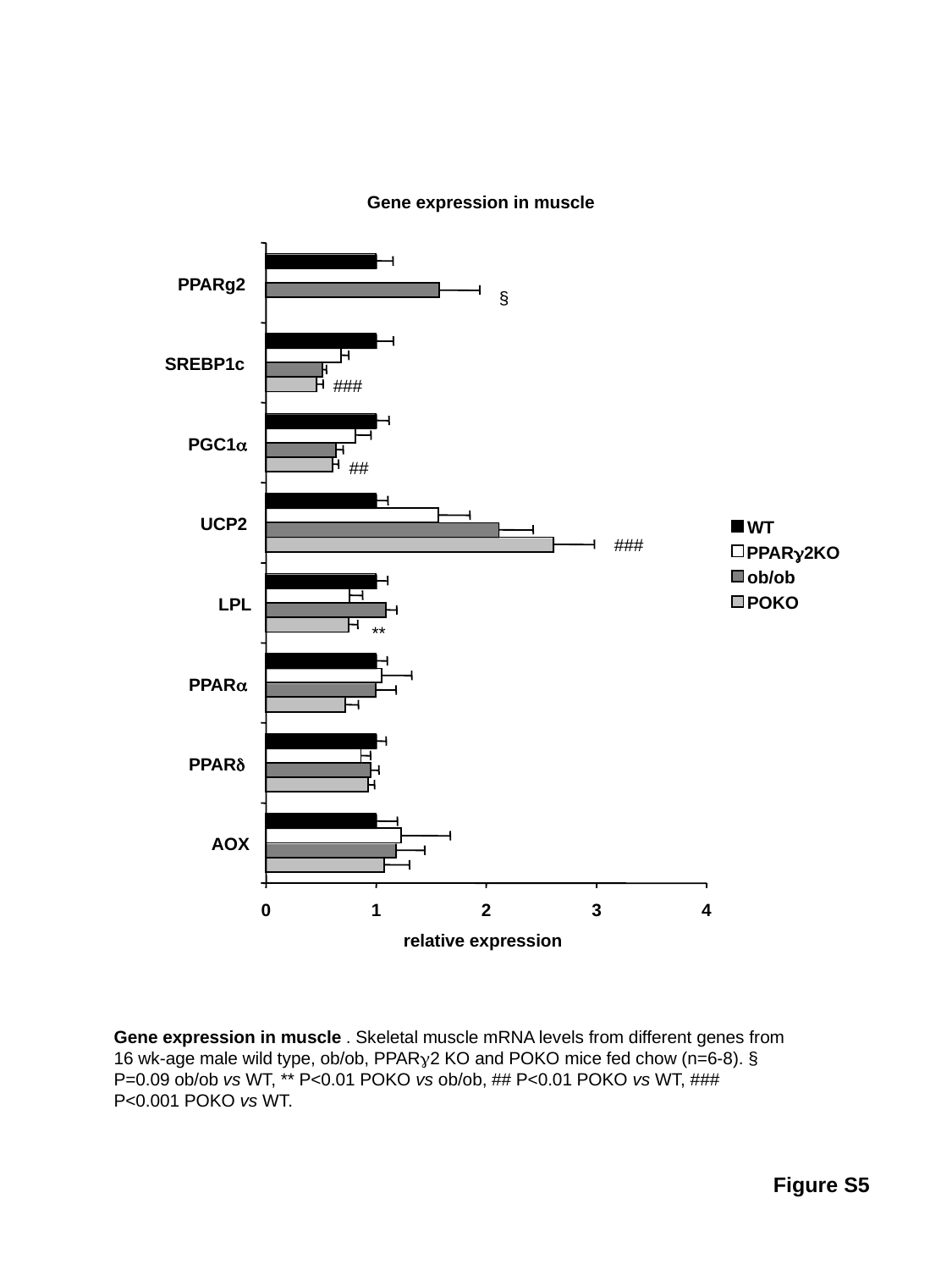

Gene expression in muscle
PPARg2
§
SREBP1c
###
PGC1α
##
UCP2
WT
###
PPARγ2KO
ob/ob
POKO
LPL
**
PPARα
PPARδ
AOX
0
1
2
3
4
relative expression
Gene expression in muscle . Skeletal muscle mRNA levels from different genes from 16 wk-age male wild type, ob/ob, PPARγ2 KO and POKO mice fed chow (n=6-8). § P=0.09 ob/ob vs WT, ** P<0.01 POKO vs ob/ob, ## P<0.01 POKO vs WT, ### P<0.001 POKO vs WT.
Figure S5
