# Supplementary material for: PPAR gamma 2 Prevents Lipotoxicity by Controlling Adipose Tissue Expandability and Peripheral Lipid Metabolism
Source: PLoS Genet. 2007 Apr 27;3(4):e64. doi: 10.1371/journal.pgen.0030064 (PMC1857730; doi:10.1371/journal.pgen.0030064)

Protocol S1: POKO mouse model

Lipidomics dataset

Page

Lipidomics dataset S1-WAT: **6**
(A) Partial least squares discriminant scores in WAT
(B) Partial least squares discriminant loads in WAT

Lipidomics dataset S2-WAT: **7 and 8**
Lipidomic profiling of WAT (selected).

Lipidomics dataset S3-Islets: **9**
(A) Partial least squares discriminant scores in pancreatic islets
(B) Partial least squares discriminant loads in pancreatic islets

Lipidomics dataset S4-Islets: **10**
Box plots for select lipids from profiling of isolated pancreatic islets

Lipidomics dataset S5-Liver: **11**
(A) Partial least squares discriminant scores in liver
(B) Partial least squares discriminant loads in liver

Lipidomics dataset S6-Liver: **12**
Liver lipidomics results for sphingolipids and lysophosphtidylcholines

Lipidomics dataset S7-Liver: **13**
Ceramide profiles in liver in POKO and ob/ob genotypes

Lipidomics dataset S8-liver: **14**
(A) Liver short and medium chain triacylglycerols profiling results
(B) Levels of diacylglycerols in liver

Lipidomics dataset S9-liver: **15**
Levels of phosphatidylcholines

Lipidomics dataset S10-skeletal muscle: **16**
(A) Partial least squares discriminant scores in skeletal muscle
(B) Partial least squares discriminant loads in skeletal muscle

Lipidomics dataset S11- skeletal muscle: **17**
Lipid profiles of identified lipids in skeletal muscle

Lipidomics dataset S12- skeletal muscle: **18**
Box plots for specific lipids in skeletal muscle

Lipidomics dataset S13: **19**
Box plots for added intensities of short-, medium-, and long-chain triacylglycerols in liver.
Lipidomics dataset S14: **20**
Median levels of individual TAG molecular species in liver

Figure captions

Note1: The box plots should be interpreted as follows: The upper edge (hinge) of the box indicates the 75th percentile of the data set, and the lower hinge indicates the 25th percentile. The line in the box indicates the median value of the data. The ends of the vertical lines or "whiskers" indicate the minimum and maximum data values, unless outliers are present in which case the whiskers extend to a maximum of 1.5 times the inter-quartile range. The points outside the ends of the whiskers are outliers or suspected outliers.

Note 2: Partial least squares discriminant analysis (PLS/DA) [1,2] was utilized as a supervised modeling method using SIMPLS algorithm to calculate the model [3]. PLS/DA is supervised pattern recognition technique that correlates variation in the dataset with class membership. Specifically, PLS analysis maximizes the product of variance matrix of measured variables (e.g. lipid profile data) and correlation of measured data with properties of interest (e.g. different genotypes). The resulting projection model gives latent variables (LVs) that focus on maximum separation (“discrimination’’). PLS/DA has been widely used in genomic and metabolomic studies (see for example [4,5]). As the total number of samples was insufficient for independent validation, no hold-out dataset was utilized for cross-validation. Instead, Venetian blinds cross-validation method [6] and $Q2$ scores were used to optimize the model, where parameter $Q2$ is a measure of the predictive ability of the model. Top loadings for latent variables (LVs) associated with drug specific effects were reported. Multivariate analyses were performed using Matlab version 7.2 (Mathworks, Inc.) and the PLS Toolbox version 3.5 Matlab package (Eigenvector Research, Inc.).

Caption Lipidomics dataset S1: (A) Partial least squares discriminant analysis of POKO, ob/ob and WT mice reveals three distinct lipid phenotypes in WAT. (B) The POKO phenotype is correlated mainly with diacylglycerols. The POKO and ob/ob phenotypes are associated with two ceramide and three lysophosphatidylcholine species (Figure Lipids2)

Caption Lipidomics dataset S2: Lipidomic profiling of WAT (selected). (A) Diacylglycerols (DAG) are upregulated in POKO relative to WT, and also relative to ob/ob for less saturated DAGs. Short chain triacylglycerols are downregulated in POKO. Long chain TAGs are downregulated in ob/ob. The *p*-values are based on 3-group comparison using one-way ANOVA.(B) The subtle level increase in ceramide species and LysoPC species is, expect for LysoPC(18:1), not statistically significant. Both POKO and ob/ob have sharply decreased levels of sphingomyelin(d18:1/16:0) and ethanolamine plasmalogen (36:1). Same trend was observed for other sphingomyelins. The *p*-values are based on 3-group comparison using one-way ANOVA.

Caption Lipidomics dataset S3: Lipidomic profiling of isolated pancreatic islets in POKO(3 mice), PPARg2 KO (3 mice), and WT (5 mice). (A) Patterns specific to different genotypes is apparent, particularly for the WT and POKO. (B) Notable associations are the two ceramides (20:0 and 22:0 fatty acids) with the POKO phenotype and the differences in triacylglycerol distribution. There is also evident phospholipid redistribution, most notably upregulation of phosphatidylethanolamine (36:2) at expense of ethanolamine plasmalogen (36:2) in POKO mice (see also Figure Lipids4).

Caption Lipidomics dataset S4: Box plots for select lipids from profiling of isolated pancreatic islets in POKO (3 mice), PPARg2 KO (3 mice), and WT (5 mice). The *p*-values are based on 3-group comparison using one-way ANOVA.

Caption Lipidomics dataset S5: Lipidomic profiling of liver. (A) PLS/DA score plot of reveals large liver lipid profile between the POKO, ob/ob, and WT genotypes. No significant changes were found between the WT and PPARg2 KO genotypes (this also holds for the other discriminant components). (B) PLS/DA loads demonstrate correlations of ceramides and long chain triacylglycerols with the POKO genotype. Only the relevant subset of negative values of second latent variables is shown (LV2).

Caption Lipidomics dataset S6: Liver lipidomics results for sphingolipids and lysophosphtidylcholines. (A) Upregulation of ceramides is observed in POKO and ob/ob (see also Figure Lipids7), while no such trend is observed for sphingomyelins. (B) Increased levels of lysophosphatidylcholines in POKO and ob/ob mouse livers are observed. The fold changes are calculated relative to the median intensity of the WT profiles.

Caption Lipidomics dataset S7: Ceramide profiles in liver show common pattern of upregulation in POKO and ob/ob genotypes. The ceramide levels, except for d18:1/24:1, are generally higher in POKO than in ob/ob mice.

Caption Lipidomics dataset S8: Liver lipid profiling results. (A) Increased levels of short and medium chain triacylglycerols are observed in ob/ob mice. These triacylglycerols are also increased in POKO mice relative to WT, but less than in ob/ob. The long chain triacylglycerols are increased only in POKO mice. (B) Increased levels of diacylglycerols are observed only in the ob/ob mice. The fold changes are calculated relative to the median intensity of the WT profiles.

Caption Lipidomics dataset S9: Downregulation of several abundant phosphatidylcholines is observed in POKO mice.

Caption Lipidomics dataset S10: (A) Multivariate analysis of skeletal muscle lipidomics data for POKO, ob/ob, and WT mouse models. (B) The changes specific to POKO genotypes are correlated with increased ceramide (d18:1/18:0), lysophosphatidylcholines, diacylglycerols, and with decrease in short chain triacylglycerols.

Caption Lipidomics dataset S11: Lipid profiles of identified lipids in skeletal muscle, excluding phosphatidylcholines, -ethanolamines, and -serines.

Caption Lipidomics dataset S12: Box plots for specific lipids in skeletal muscle. The changes are less pronounced as in liver, with the most significant change being the POKO-specific downregulation of short chain triacylglycerols. Subtle increase of ceramide (d18:1/18:0) is observed.

Caption Lipidomics dataset S13: Box plots for added intensities of short-, medium-, and long-chain triacylglycerols in liver. Note the log-scale used. Medium chain TAGs are at least two orders of magnitude more abundant than long- and short-chain TAG species, so they are the major components contributing to total TAG measure.

Caption Lipidomics dataset S14: Median levels of individual TAG molecular species in liver.

References:

1. Geladi P, Kowalski BR (1986) Partial least-squares regression: a tutorial. Anal Chim Acta 185: 1-17.
2. Barker M, Rayens W (2003) Partial least squares for discrimination. J Chemometrics 17: 166-173.
3. de Jong S (1993) SIMPLS: An alternative approach to partial least squares regression. Chemometr Intell Lab Syst 18: 251-263.
4. Perez-Enciso M, Tenenhaus M (2003) Prediction of clinical outcome with microarray data: a partial least squares discriminant analysis (PLS-DA) approach. Hum Genet 112: 581-592.
5. Eriksson L, Antti H, Gottfries J, Holmes E, Johansson E, et al. (2004) Using chemometrics for navigating in the large data sets of genomics, proteomics, and metabonomics (gpm). Anal Bioanal Chem 380: 419-429.
6. Wise BM, Gallagher NB, Bro R, Shaver JM, Windig W, et al. (2005) PLS Toolbox 3.5 for use with Matlab. Manson, WA: Eigenvector Research Inc.

Lipidomics dataset S1-WAT

(A)

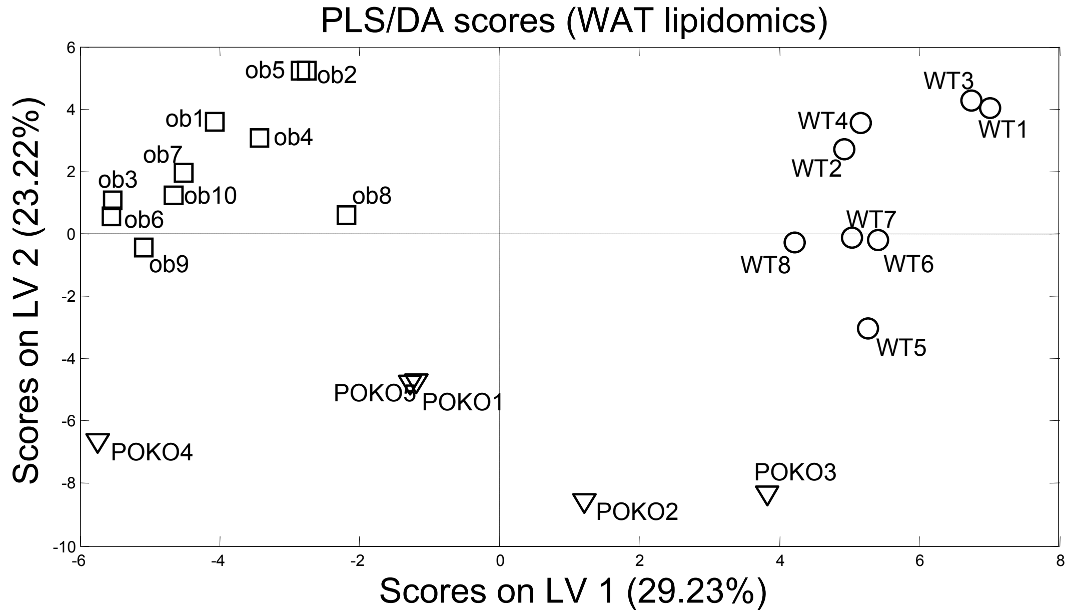

(B)

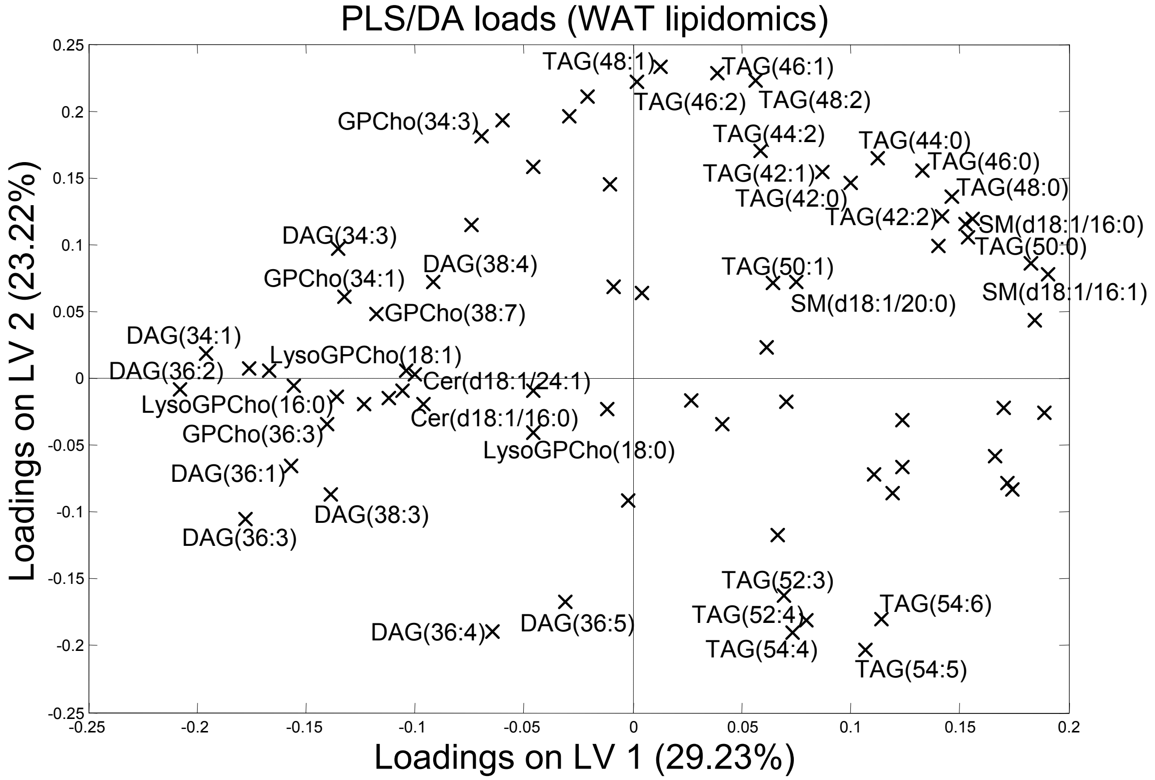

Lipidomics dataset S2A-WAT

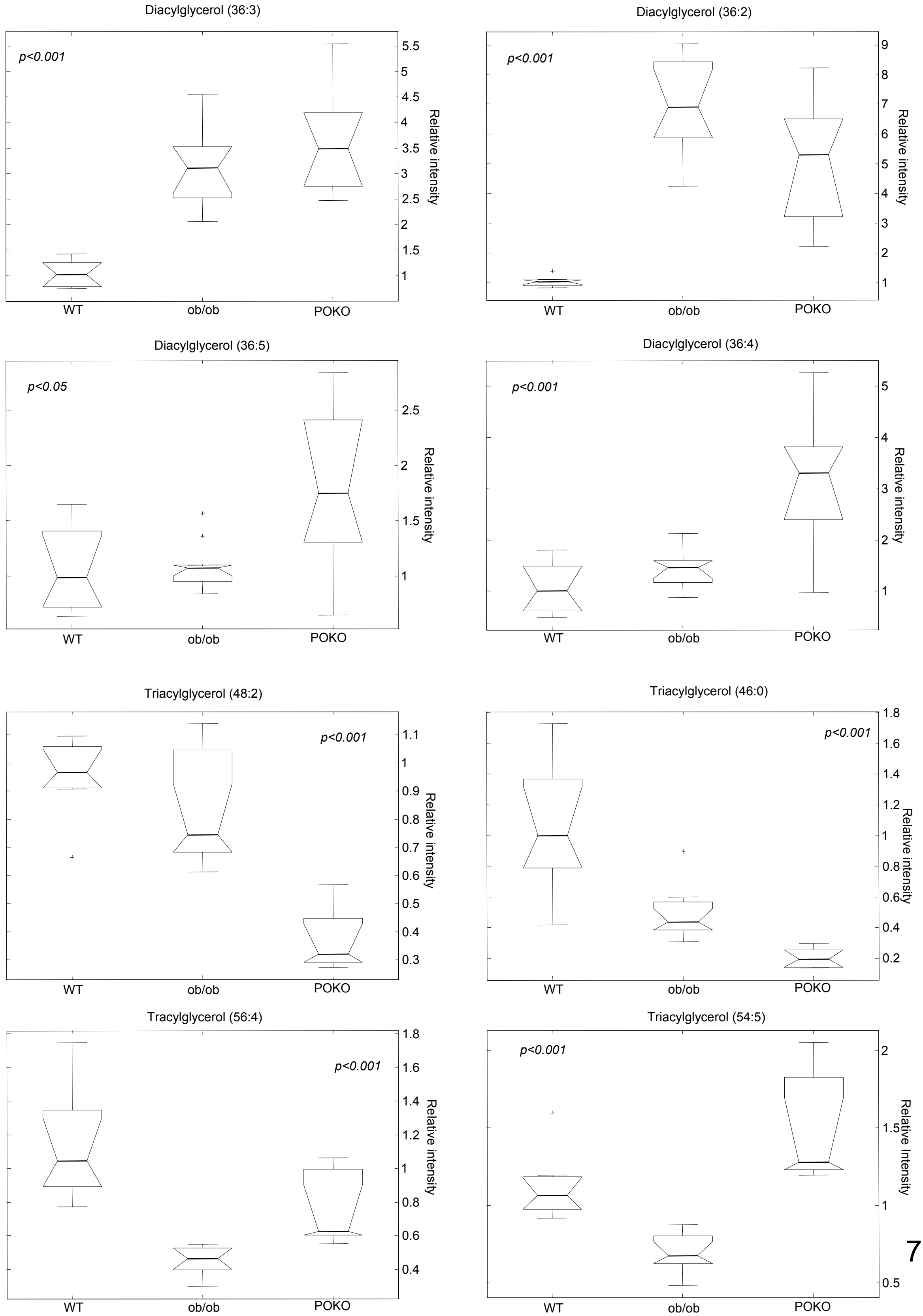

Lipidomics dataset S2B- WAT

Ceramide (d18:1/24:1)

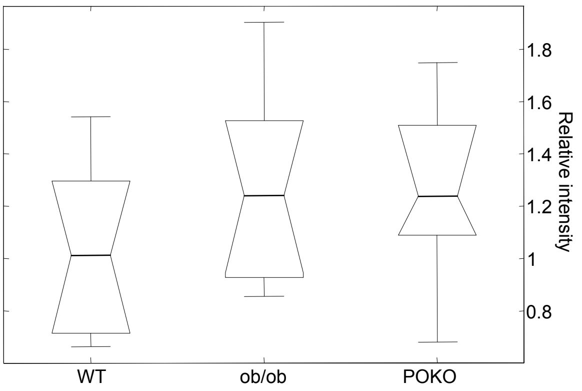

Ceramide (d18:1/16:0)

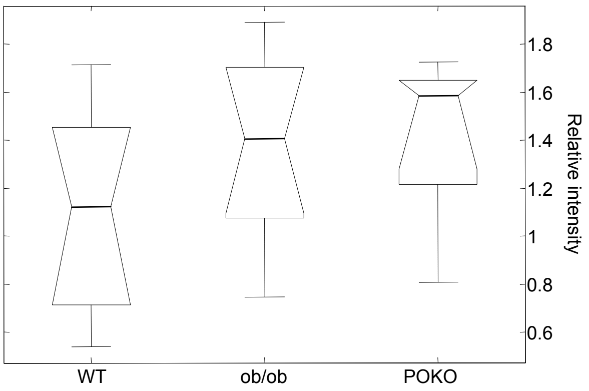

Lysophosphatidylcholine (18:1)

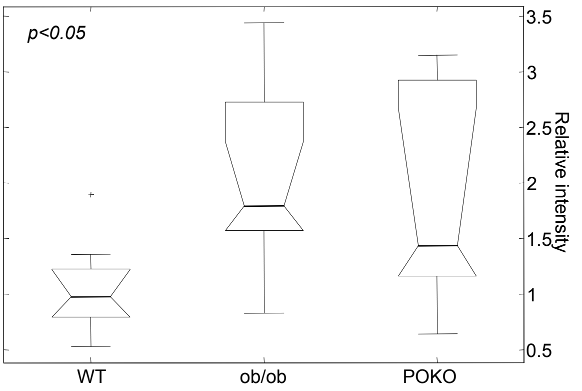

Lysophosphatidylcholine (18:0)

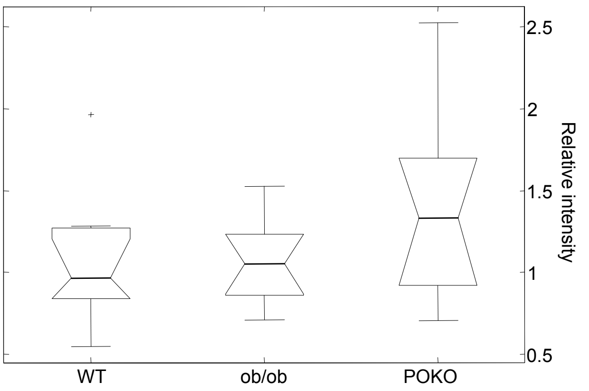

Ethanolamine plasmalogen (36:1)

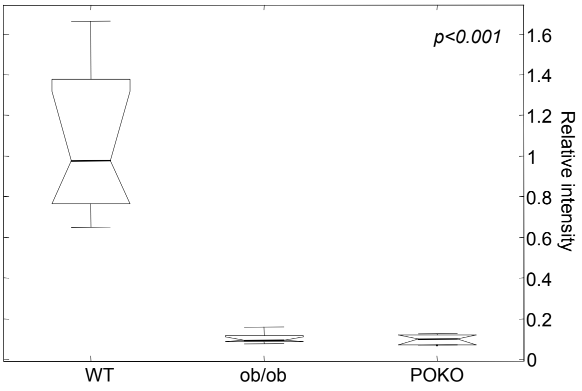

Sphingomyelin (d18:1/16:0)

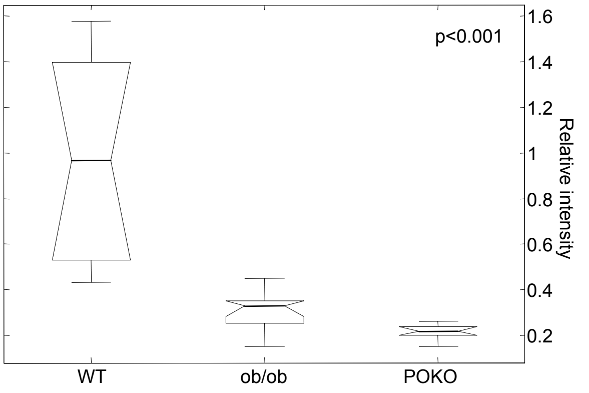

Lipidomics dataset S3- ISLETS

(A)

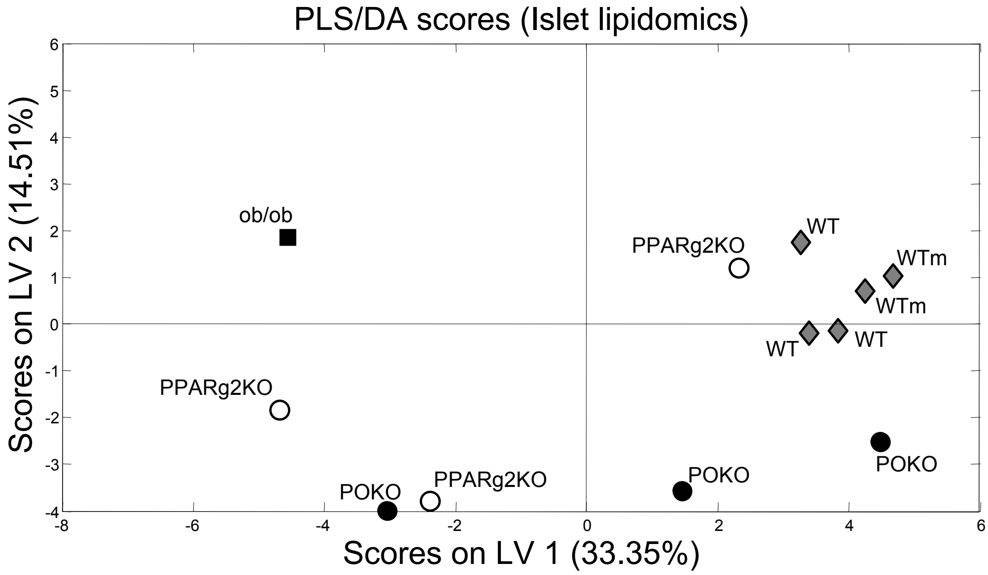

(B)

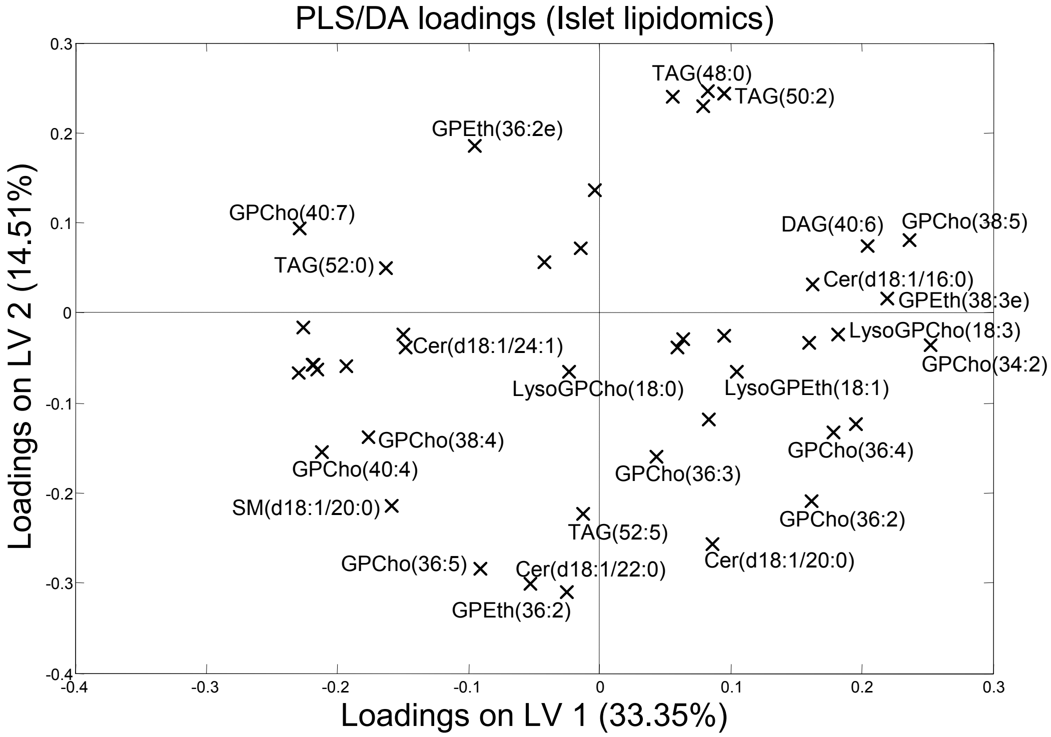

Lipidomics dataset S4-ISLETS

Ceramide (d18:1/22:0)

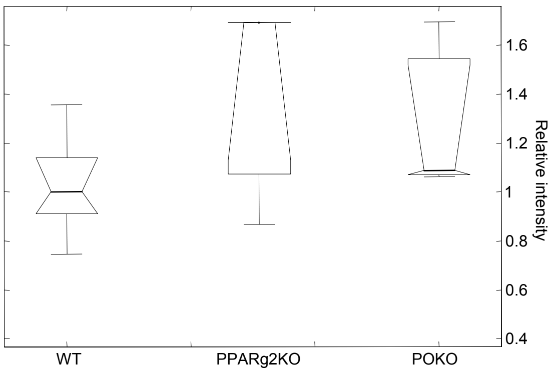

Ceramide (d18:1/20:0)

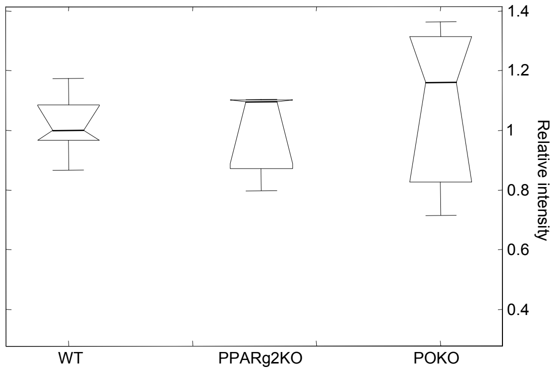

Phosphatidylethanolamine (36:2)

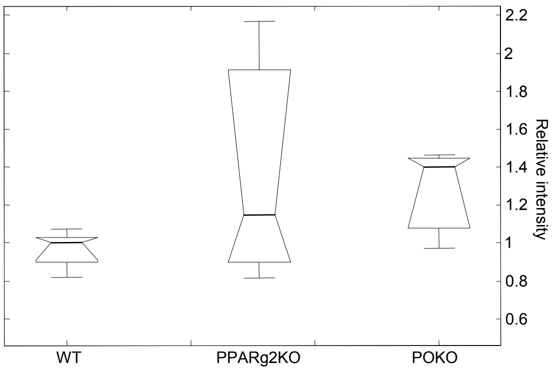

Ethanolamine plasmalogen (36:2)

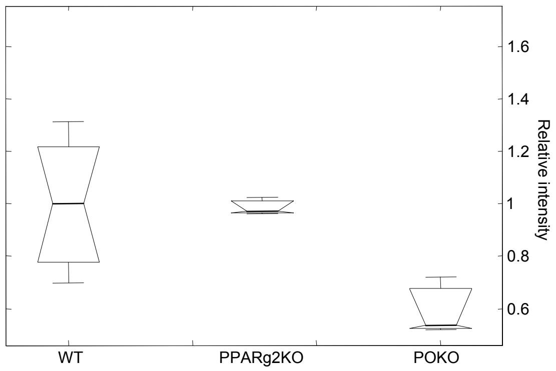

Phosphatidylcholine (36:2)

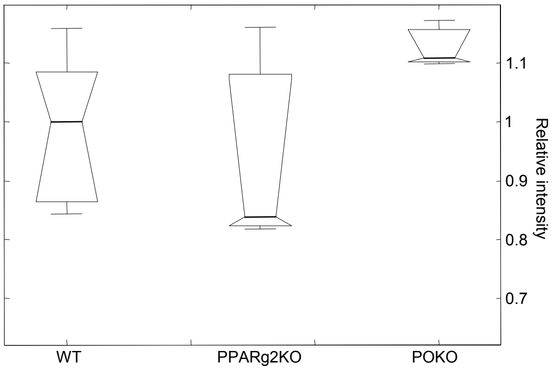

Phosphatidylcholine (34:2)

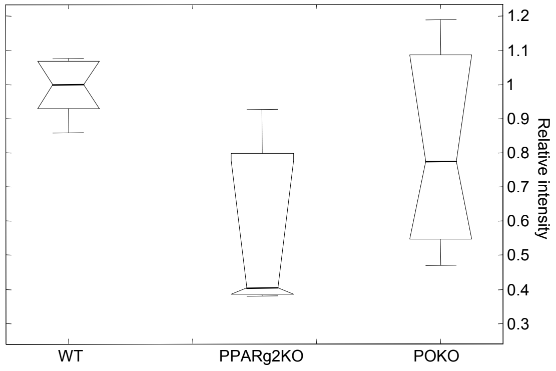

Triacylglycerol (52:5)

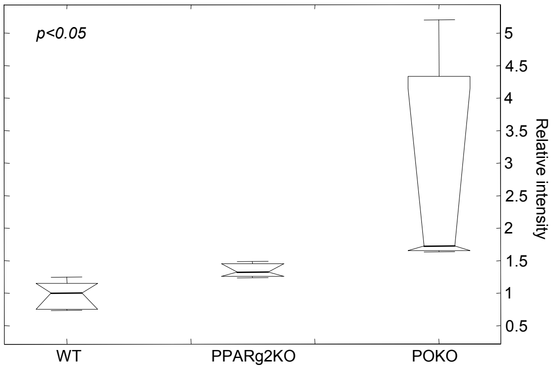

Triacylglycerol (48:0)

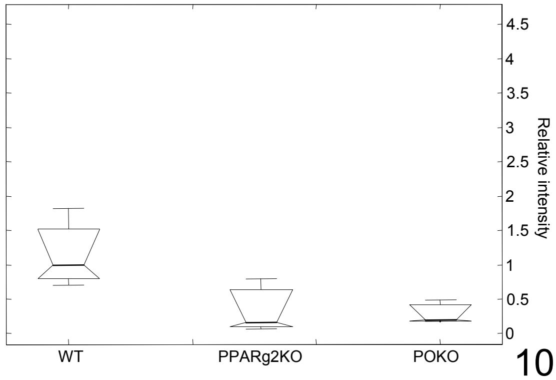

Lipidomics dataset S5-LIVER

(A)

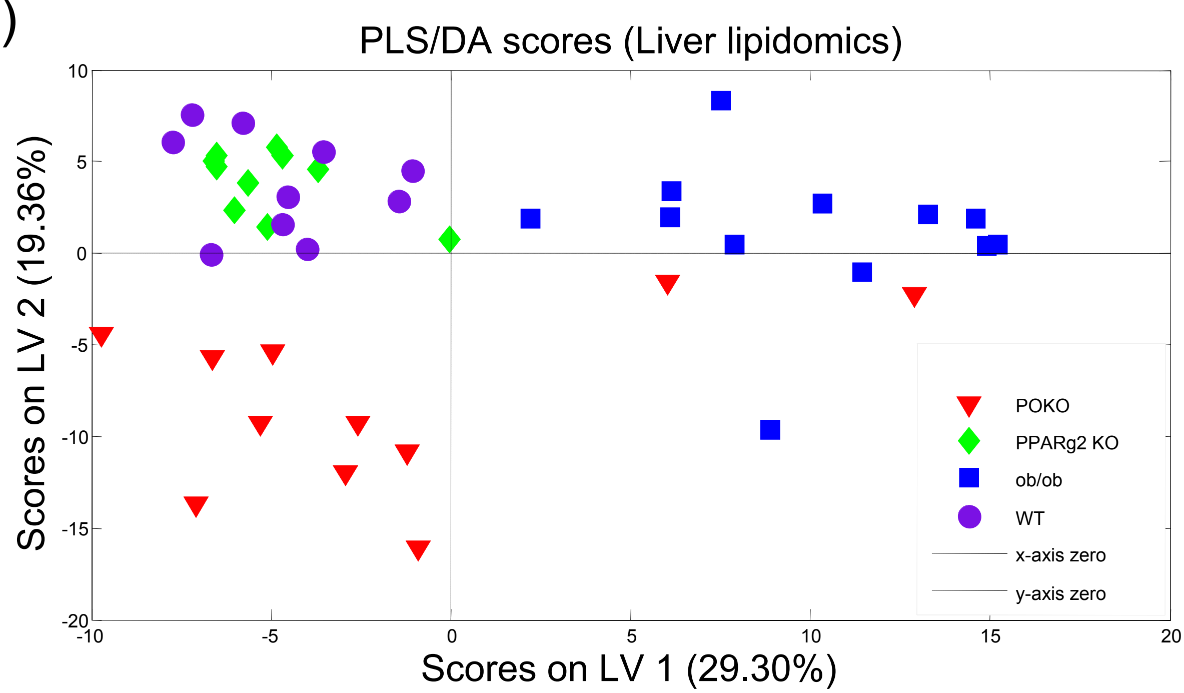

(B)

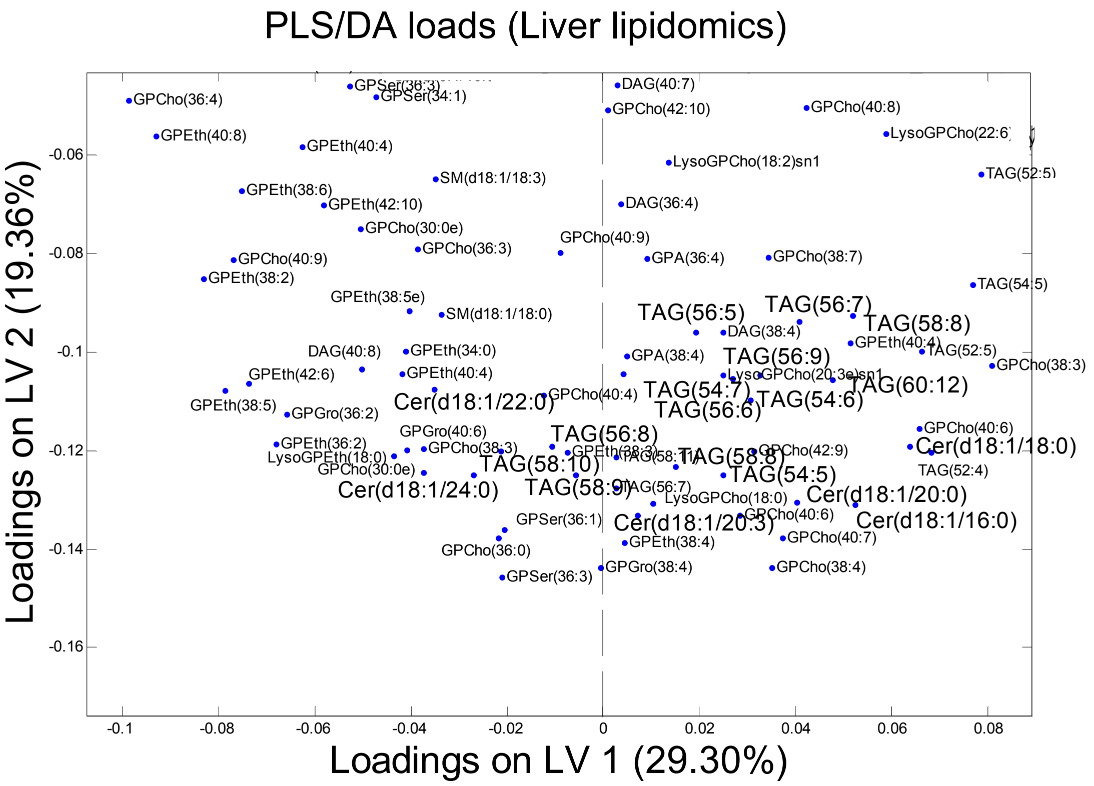

Lipidomics dataset S6-LIVER

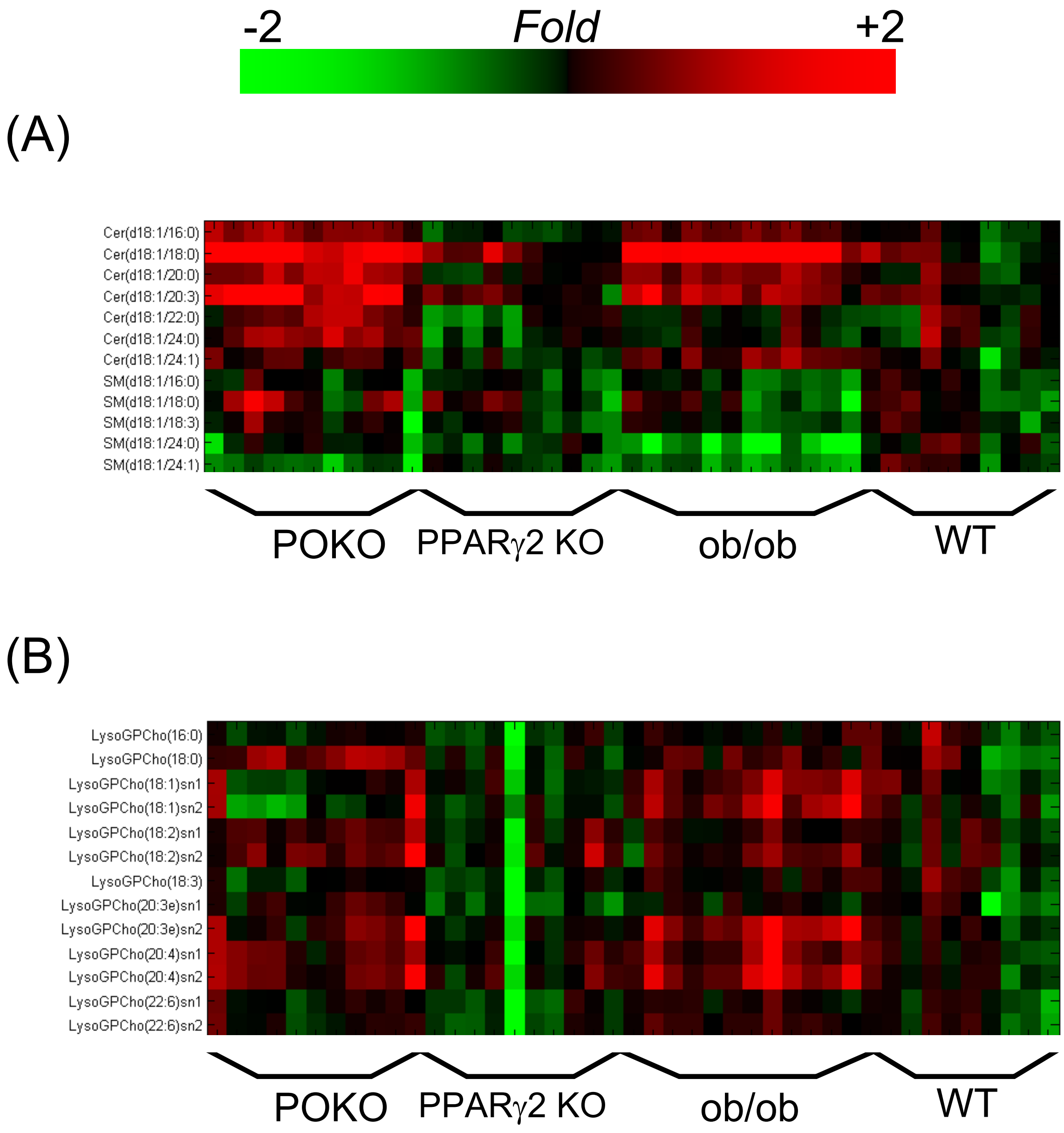

Lipidomics dataset S7-LIVER

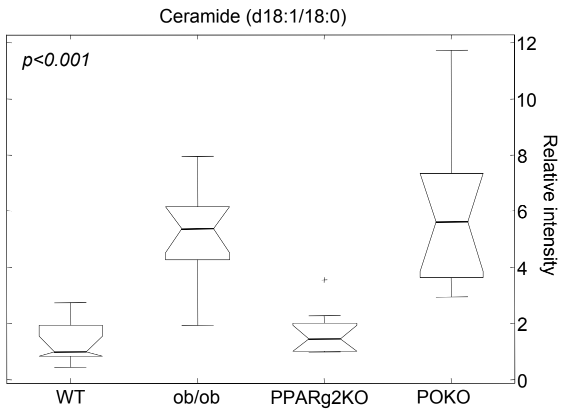

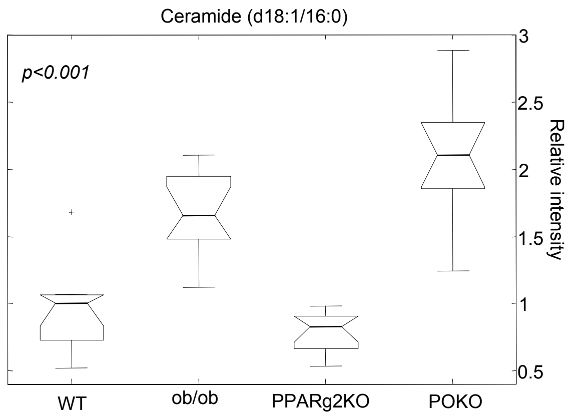

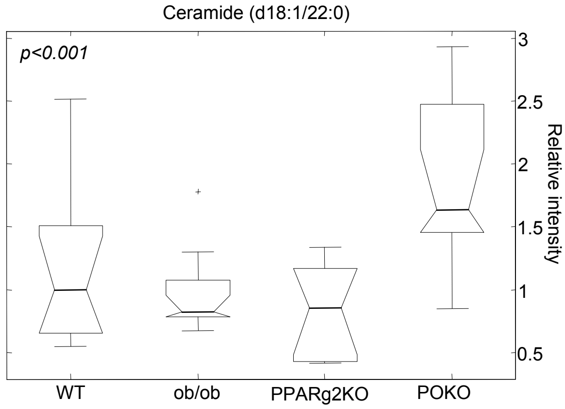

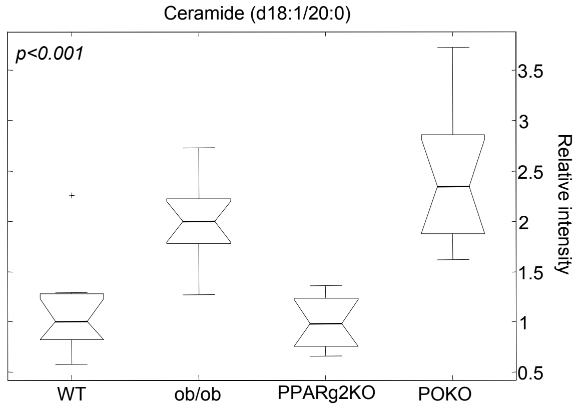

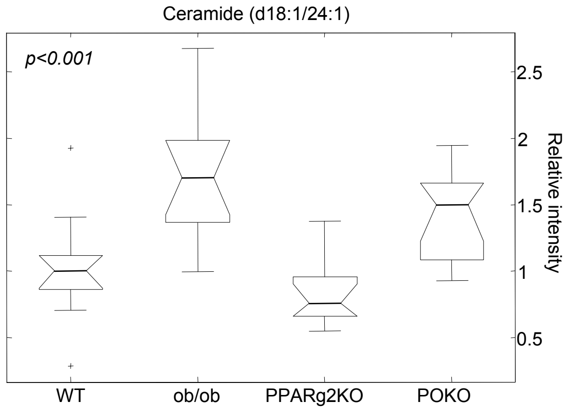

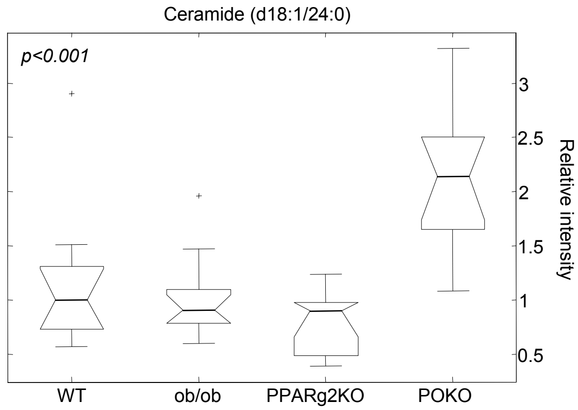

Lipidomics dataset S8-LIVER

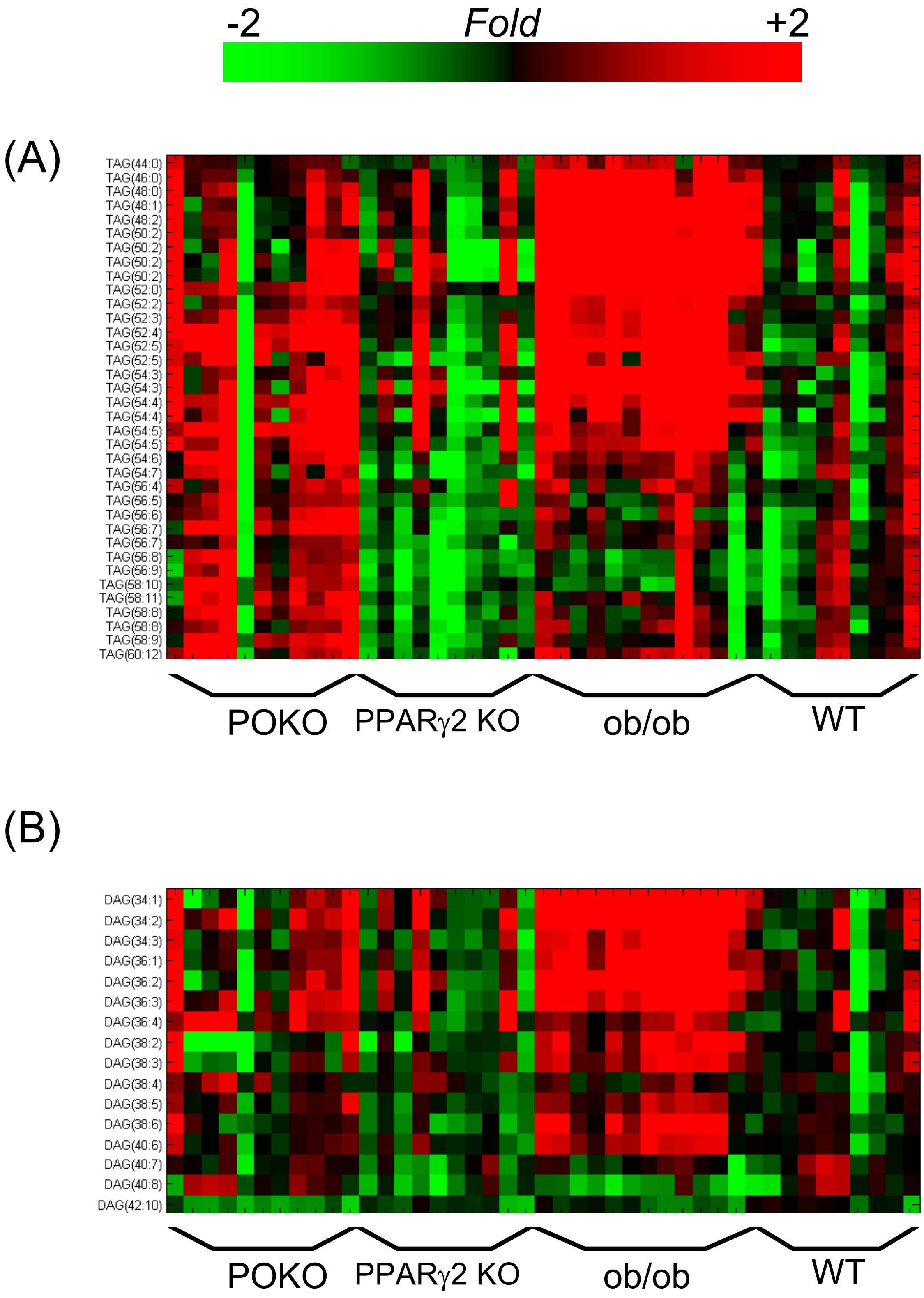

Lipidomics dataset S9-LIVER

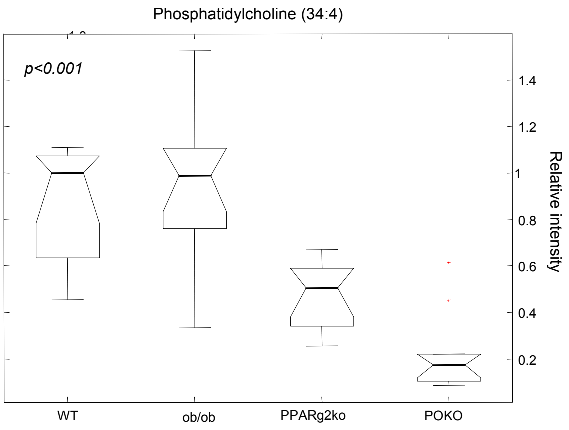

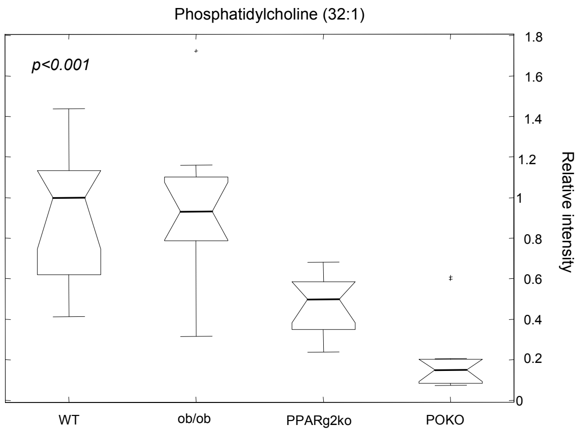

Lipidomics dataset S10-Skeletal muscle

(A)

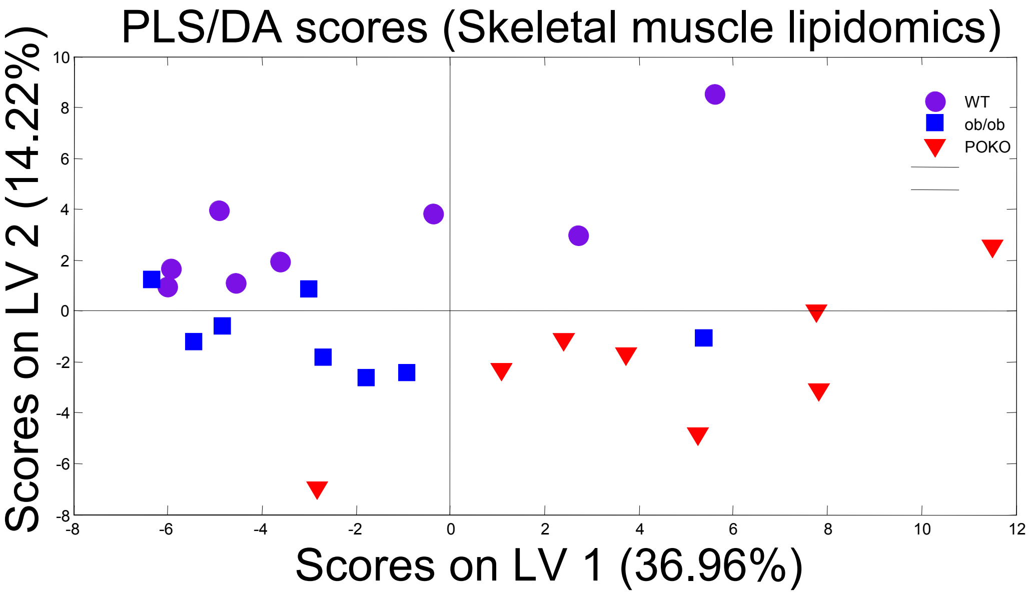

(B)

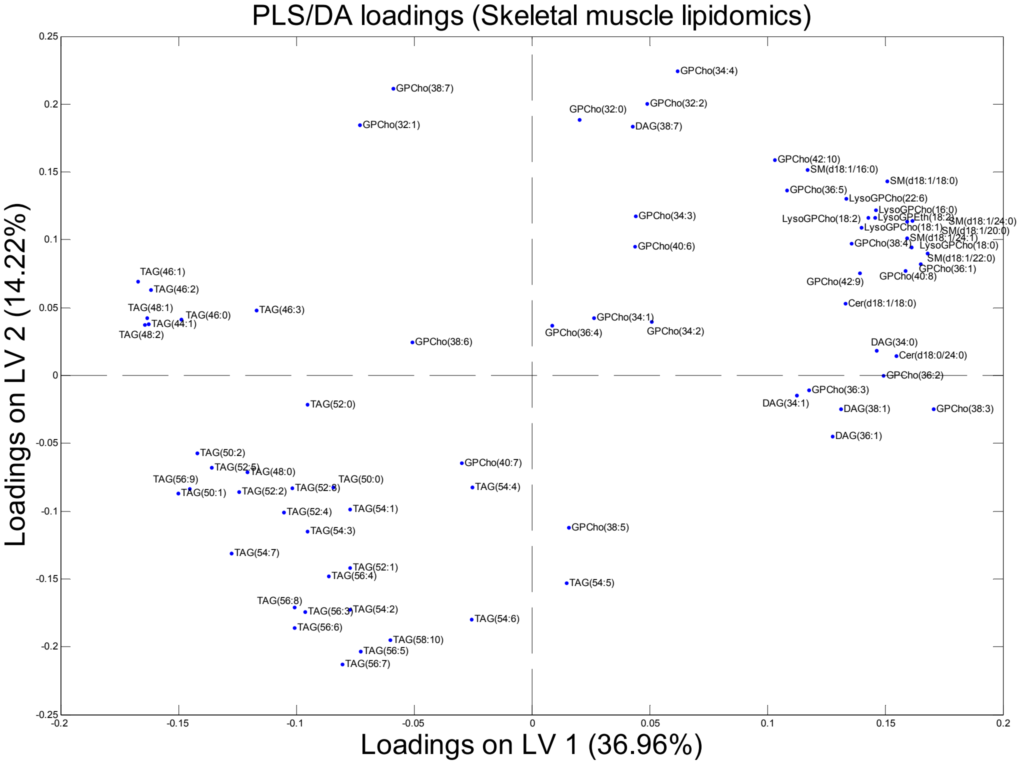

Lipidomics dataset S11-Skeletal muscle

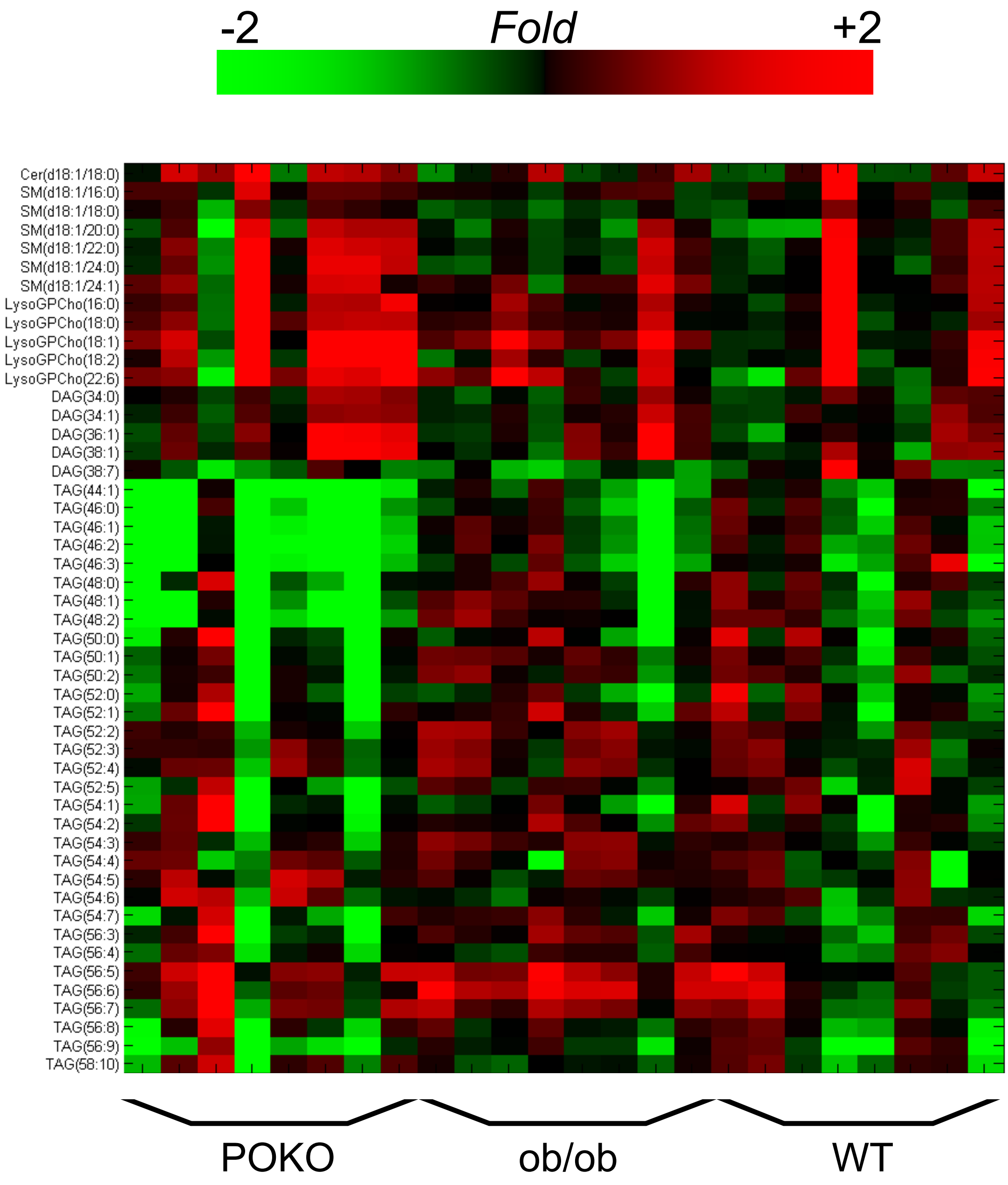

Lipidomics dataset S12-Skeletal muscle

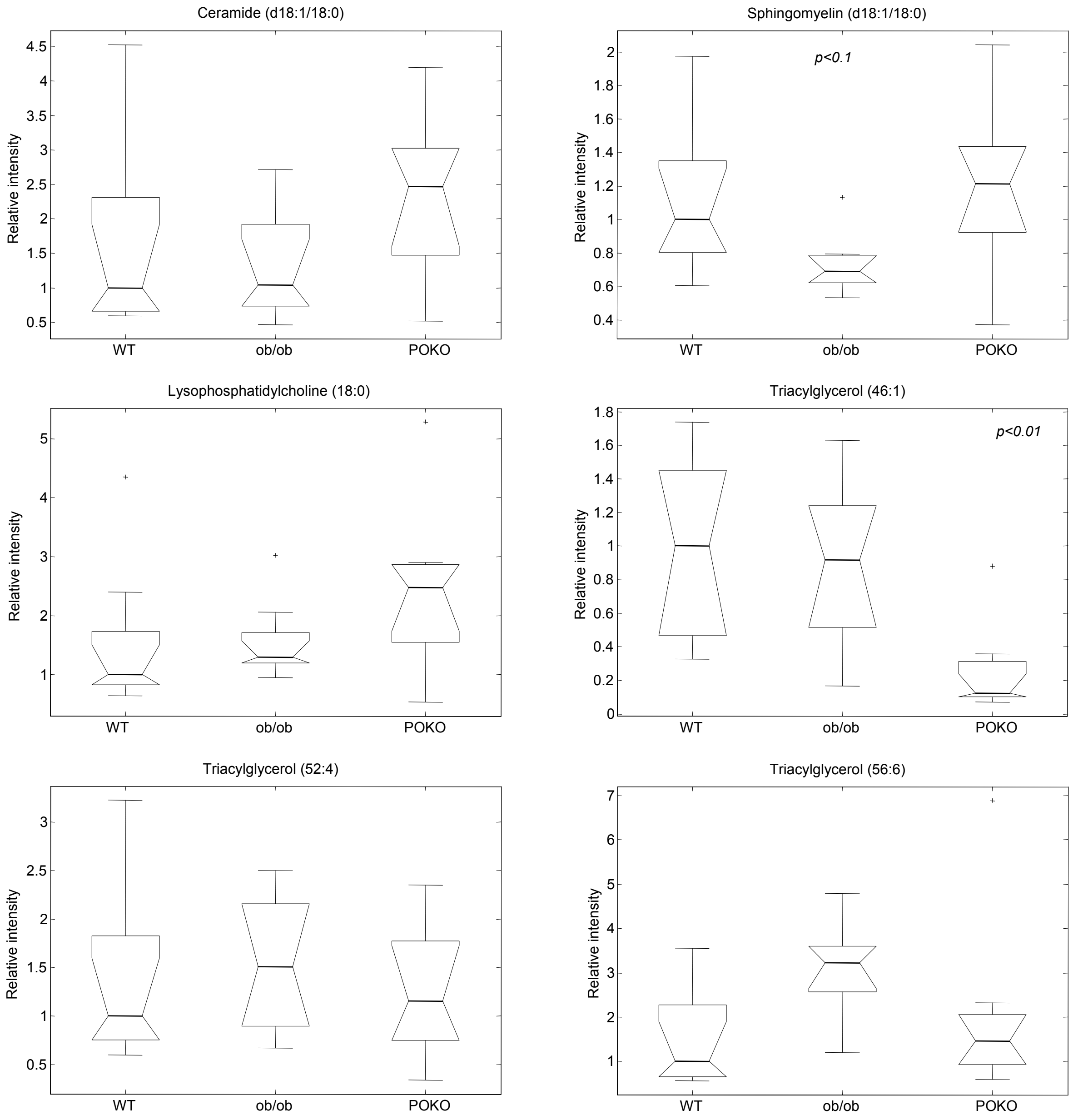

Lipidomics dataset S13-LIVER

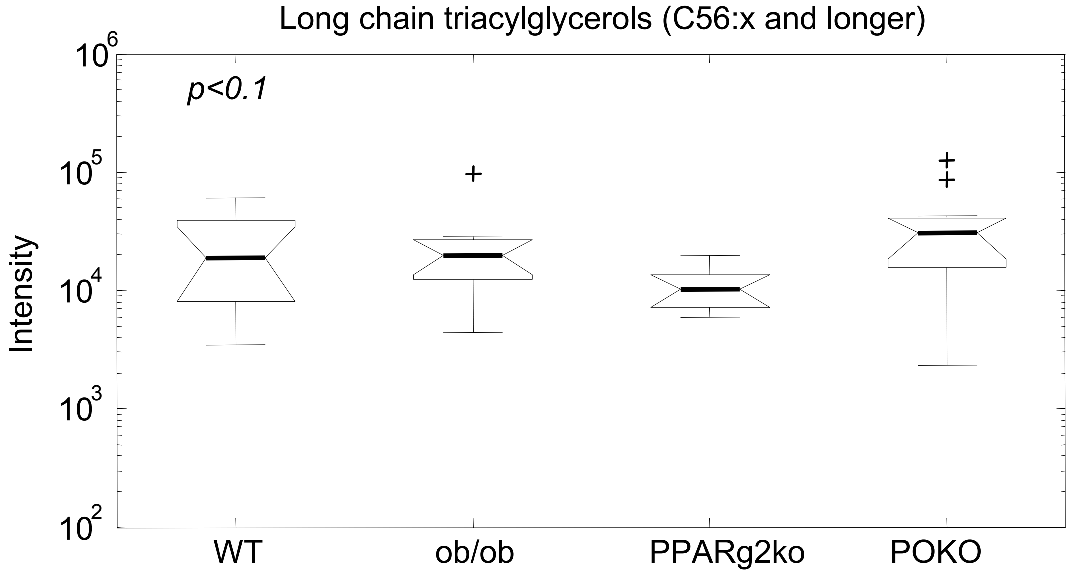

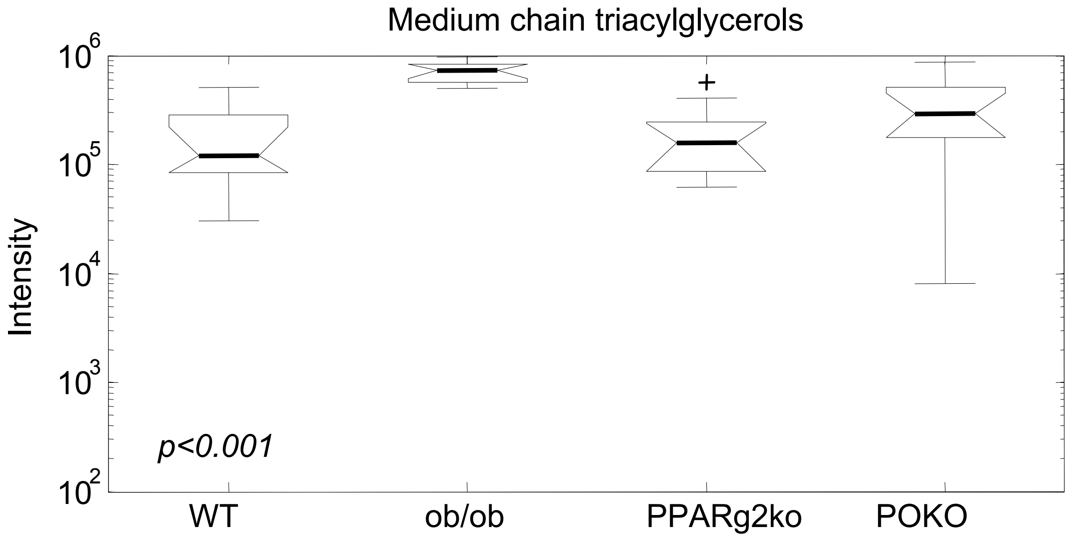

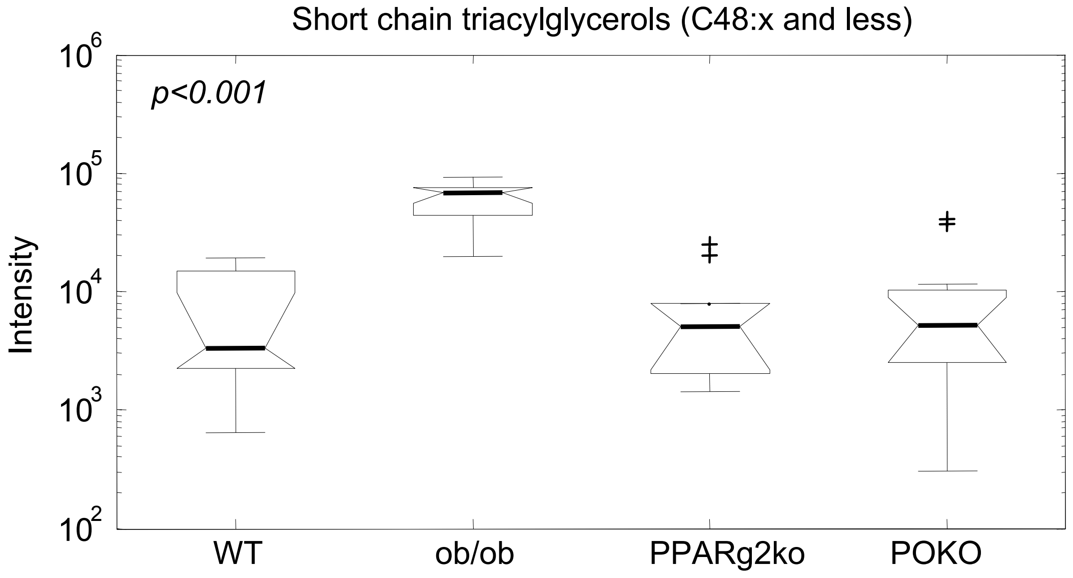

Lipidomics dataset S14-LIVER

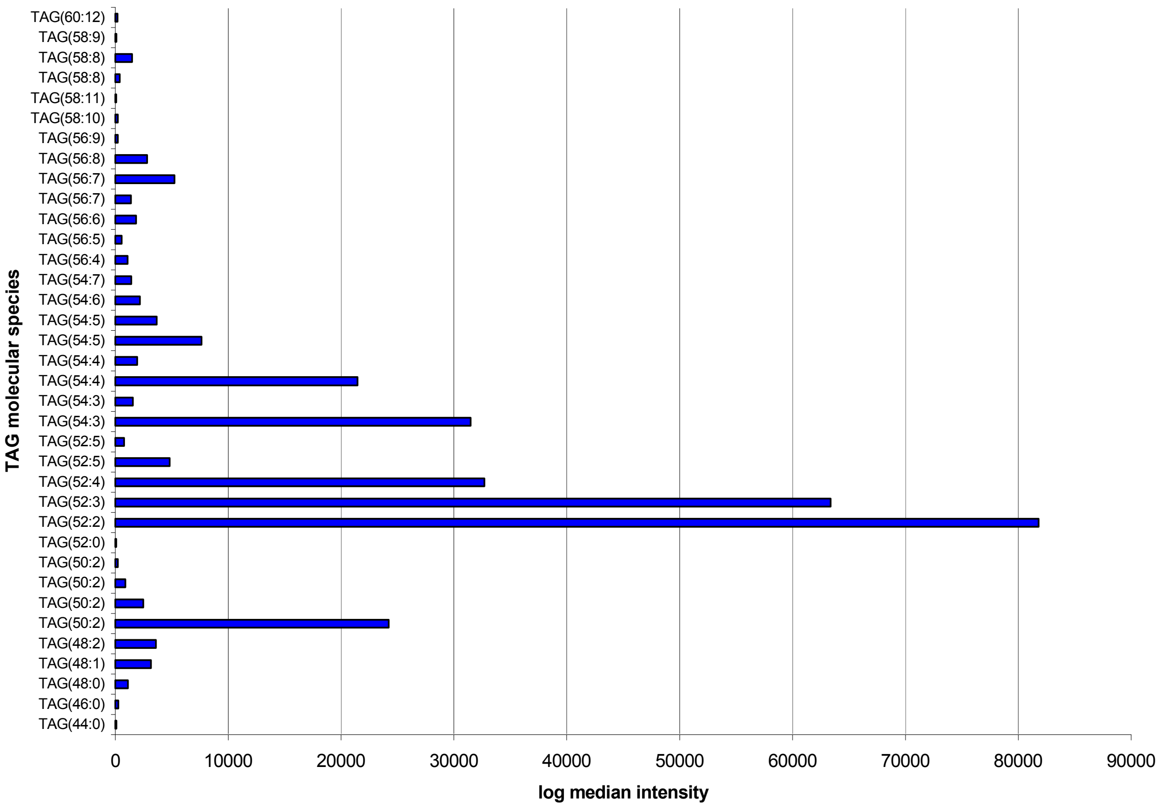

Supplement: Protocol S1 — (208 KB PDF) [file pgen.0030064.sd001.pdf]
