# Supplementary material for: PPAR gamma 2 Prevents Lipotoxicity by Controlling Adipose Tissue Expandability and Peripheral Lipid Metabolism
Source: PLoS Genet. 2007 Apr 27;3(4):e64. doi: 10.1371/journal.pgen.0030064 (PMC1857730; doi:10.1371/journal.pgen.0030064)
Supplement: Table S1 — (29 KB PPT) [file pgen.0030064.st001.ppt]

## Slide 1
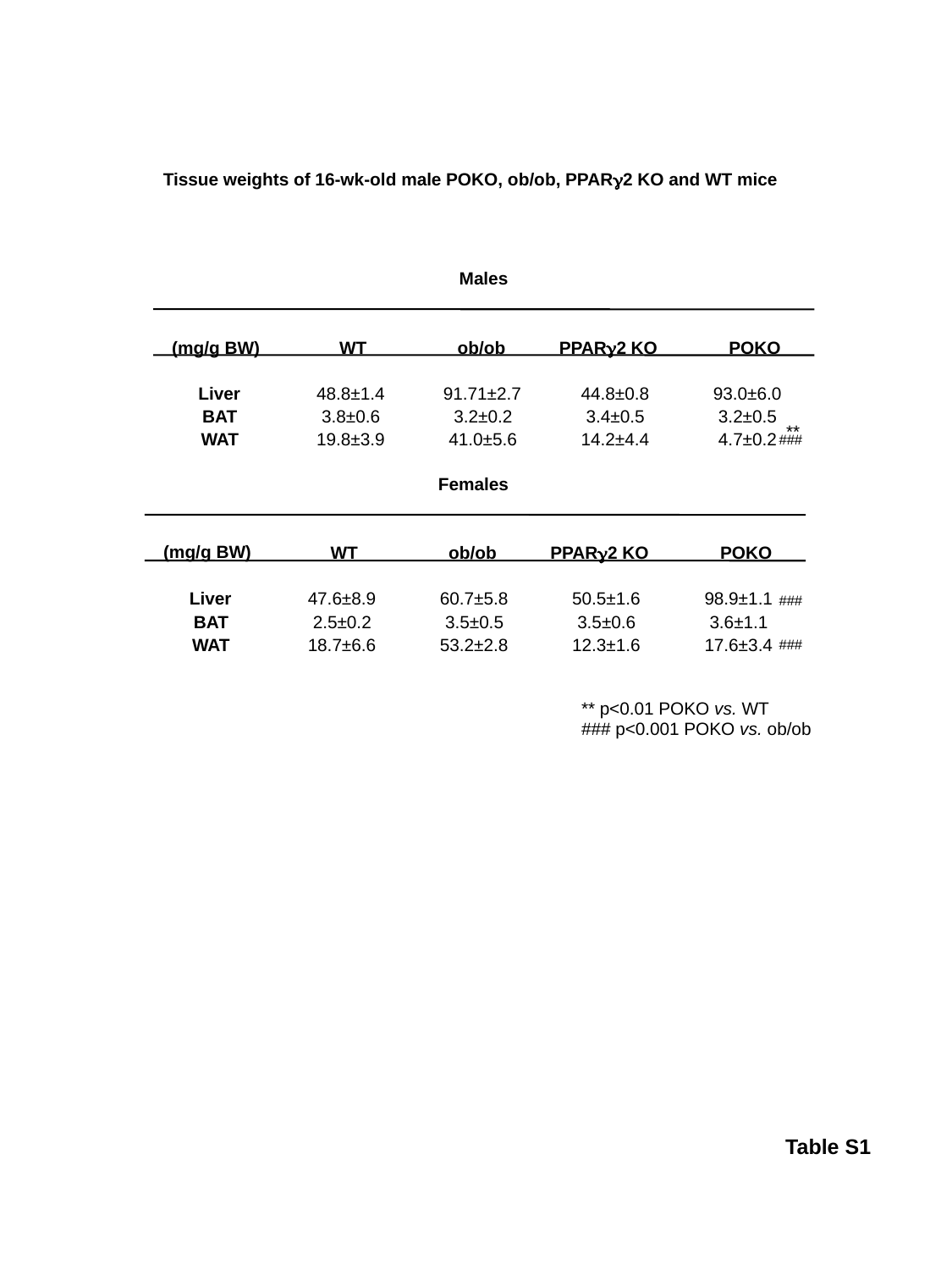

Tissue weights of 16-wk-old male POKO, ob/ob, PPARγ2 KO and WT mice
Males
(mg/g BW)
WT
ob/ob
PPARγ2 KO
POKO
Liver
48.8±1.4
91.71±2.7
44.8±0.8
93.0±6.0
BAT
3.8±0.6
3.2±0.2
3.4±0.5
3.2±0.5
**
###
WAT
19.8±3.9
41.0±5.6
14.2±4.4
4.7±0.2
Females
WT
ob/ob
PPARγ2 KO
POKO
###
Liver
47.6±8.9
60.7±5.8
50.5±1.6
98.9±1.1
BAT
2.5±0.2
3.5±0.5
3.5±0.6
3.6±1.1
###
WAT
18.7±6.6
53.2±2.8
12.3±1.6
17.6±3.4
** p<0.01 POKO vs. WT
### p<0.001 POKO vs. ob/ob
(mg/g BW)
Table S1
