# Supplementary material for: PPAR gamma 2 Prevents Lipotoxicity by Controlling Adipose Tissue Expandability and Peripheral Lipid Metabolism
Source: PLoS Genet. 2007 Apr 27;3(4):e64. doi: 10.1371/journal.pgen.0030064 (PMC1857730; doi:10.1371/journal.pgen.0030064)
Supplement: Table S2 — (105 KB DOC) [file pgen.0030064.st002.doc]

| Table S2: Microarray data | | | | | | | | |
| --- | --- | --- | --- | --- | --- | --- | --- | --- |
| Tissue | WAT | | liver | | muscle | |  |  |
| Genbank Accession | ratio | p | ratio | p | ratio | p | Common name | description |
| NM_009204 | 1.08 | 0.852 | 0.67 | 0.365 | 1.17 | 0.773 | Slc2a4; glut4 | solute carrier family 2 (facilitated glucose transporter), member 4 |
| NM_013459 | **0.33** | **0.038** | 1.05 | 0.792 | 0.65 | 0.122 | Adn | adipsin |
| K02109 | 1.36 | 0.230 | 0.79 | 0.402 | 0.85 | 0.082 | Fabp4; aP2 | fatty acid binding protein 4, adipocyte |
| NM_011044 | **1.97** | **0.015** | **1.48** | **0.013** | 0.90 | 0.143 | Pck1 | phosphoenolpyruvate carboxykinase 1, cytosolic |
| NM_020509 | **4.30** | **0.003** | 0.81 | 0.306 | 1.09 | 0.572 | Retnla | resistin like alpha |
| AF127033 | 0.95 | 0.610 | 0.66 | 0.065 | 0.85 | 0.416 | Fasn | fatty acid synthase |
| NM_009127 | **0.43** | **0.0001** | **0.31** | **0.0001** | **0.58** | **0.00004** | Scd1 | stearoyl-Coenzyme A desaturase 1 |
| NM_011435 | **1.23** | **0.002** | 0.88 | 0.176 | 0.93 | 0.344 | Sod3 | superoxide dismutase 3, extracellular |
| NM_008161 | **2.07** | **0.001** | 1.29 | 0.232 | 0.91 | 0.394 | Gpx3 | glutathione peroxidase 3 |
| NM_008182 | 0.97 | 0.782 | 0.81 | 0.070 | **2.56** | **0.007** | Gsta2 | glutathione S-transferase, alpha 2 (Yc2) |
| NM_008183 | **1.80** | **0.001** | **0.75** | **0.044** | 0.88 | 0.226 | Gstm2 | glutathione S-transferase, mu 2 |
| NM_013541 | 1.16 | 0.097 | 1.02 | 0.910 | **1.10** | **0.014** | Gstp2 | glutathione S-transferase, pi 2 |
| NM_008185 | **1.85** | **0.010** | 0.94 | 0.722 | **1.20** | **0.0001** | Gstt1 | glutathione S-transferase, theta 1 |
| NM_008180 | **0.72** | **0.005** | 0.98 | 0.904 | 1.24 | 0.134 | Gss | glutathione synthetase |
| NM_010363 | **1.71** | **0.002** | 0.96 | 0.735 | 1.08 | 0.563 | Gstz1 | glutathione transferase zeta 1 (maleylacetoacetate isomerase) |
| NM_019946 | **1.23** | **0.046** | 0.84 | 0.132 | 1.03 | 0.740 | Mgst1 | microsomal glutathione S-transferase 1 |
| NM_009853 | **0.69** | **0.027** | **0.50** | **0.006** | 0.91 | 0.842 | Cd68 | CD68 antigen |
| NM_021460 | **0.48** | **0.001** | **0.73** | **0.042** | 0.94 | 0.409 | Lip1 | lysosomal acid lipase 1 |
| NM_013737 | **0.68** | **0.027** | 0.98 | 0.906 | 0.83 | 0.228 | Pla2g7 | phospholipase A2 group VII (platelet-activating factor acetylhydrolase, plasma) |
| NM_008867 | **0.70** | **0.035** | 0.90 | 0.390 | 1.17 | 0.470 | Pla2g1br | phospholipase A2, group IB, pancreas, receptor |
| AF233885 | 0.99 | 0.931 | 1.00 | 0.980 | **1.34** | **0.020** | Plce1 | phospholipase C, epsilon 1 |
| NM_011116 | **0.72** | **0.003** | 0.87 | 0.103 | 0.88 | 0.511 | Pld3 | phospholipase D3 |
| NM_010570 | 1.06 | 0.783 | 1.14 | 0.484 | 1.02 | 0.914 | Irs1 | insulin receptor substrate 1 |
| NM_007381 | 1.19 | 0.221 | 1.16 | 0.476 | 0.82 | 0.118 | Acadl | acetyl-Coenzyme A dehydrogenase, long-chain |
| NM_015729 | 0.94 | 0.520 | 1.02 | 0.890 | **0.76** | **0.041** | Acox1 | acyl-Coenzyme A oxidase 1, palmitoyl |

Table S2. Expression ratios and p values for the microarray, comparing mRNA in three different tissues between the POKO and the ob/ob mice. Values in bold indicate p ≤ 0.05; those in grey have p > 0.05

# Methodology for Microarrays

*Global amplification* *using template-switching PCR and labelling of cDNA probes for arrays*

cDNA was amplified from total RNA from epididymal WAT, liver and skeletal muscle of ob/ob and POKO genotypes (n=4-7) using template-switching PCR and labeled with Cy3 or Cy5 dyes as previously described [1]. The labeled products were individually purified on AutoSeq G-50 columns; then the Cy5 and Cy3 samples were pooled and ethanol precipitated.

*Hybridization of labelled probes to oligonucleotide arrays.*

The arrays utilized in this study are the Compugene Mouse Known Gene set of 7,524 oligonucleotides that were printed on glass slides by the Microarray group at the MRC-HGMP, Hinxton (http://www.hgmp.mrc.uk/Research/Microarray/index.jsp). For each set of conditions tested, duplicate and dye-swap hybridizations were performed. Labeled targets were resuspended in 30 μl of hybridization buffer (40% formamide, 5x SSC, 5x Denhardt's solution, 1 mM sodium pyrophosphate, 50 mM Tris pH 7.4, 0.1% SDS) together with 2 μg mouse Cot1 DNA (Invitrogen), denatured at 95°C for 5 min, incubated at 50°C for 5 min and then centrifuged at 13,000 r.p.m. for 5 min before being applied to the arrays. Hybridizations were performed under a coverslip at 50°C in a humidified chamber for 16 h. Following hybridization, slides were washed twice in 2x SSC for 10 min, twice in 0.1x SSC/0.1% SDS for 5 min and finally twice in 0.1x SSC for 5 min; all washes were performed at room temperature. After washing, slides were dried by centrifugation at 2000 g for 3 min.

Arrays were scanned on an Agilent G2565 scanner according to manufacturer’s instructions. Raw image data were extracted using Imagene^TM^ 5.0 software (BioDiscovery). Data were imported into GeneSpring^TM^ 6.2 (Silicon Genetics) for analysis. Normalization was performed using the Loess algorithm. Genes with ratios significantly different from 1 were identified using a t-test at the 95% confidence level.

### Reference

### 1. Petalidis, L., Bhattacharyya, S., Morris, G.A., Collins, V.P., Freeman, T.C., Lyons, P.A. (2003) Global amplification of mRNA by template-switching PCR: linearity and application to microarray analysis*. Nucleic Acids R*es 31, e142.
