# Supplementary material for: PPAR gamma 2 Prevents Lipotoxicity by Controlling Adipose Tissue Expandability and Peripheral Lipid Metabolism
Source: PLoS Genet. 2007 Apr 27;3(4):e64. doi: 10.1371/journal.pgen.0030064 (PMC1857730; doi:10.1371/journal.pgen.0030064)
Supplement: Table S4 — GenBank (http://www.ncbi.nlm.nih.gov/Genbank) accession numbers for the genes and gene products discussed in this paper. (58 KB DOC) [file pgen.0030064.st004.doc]

**Table S4.** Accession Numbers

The GenBank (http://www.ncbi.nlm.nih.gov/Genbank) accession numbers for the genes and gene products discussed in this paper are listed below.

| Common Name | Description | Genbank Accession Number |
| --- | --- | --- |
| *Slc2a4; glut4* | solute carrier family 2 (facilitated glucose transporter), member 4 | NM_009204 |
| *Adn* | adipsin | NM_013459 |
| *Fabp4; aP2* | fatty acid binding protein 4, adipocyte | K02109 |
| *Pck1* | phosphoenolpyruvate carboxykinase 1, cytosolic | NM_011044 |
| *Retnla* | resistin like alpha | NM_020509 |
| *Fasn* | fatty acid synthase | AF127033 |
| *Scd1* | stearoyl-coenzyme A desaturase 1 | NM_009127 |
| *Sod3* | superoxide dismutase 3, extracellular | NM_011435 |
| *Gpx3* | glutathione peroxidase 3 | NM_008161 |
| *Gsta2* | glutathione S-transferase, alpha 2 (Yc2) | NM_008182 |
| *Gstm2* | glutathione S-transferase, mu 2 | NM_008183 |
| *Gstp2* | glutathione S-transferase, pi 2 | NM_013541 |
| *Gstt1* | glutathione S-transferase, theta 1 | NM_008185 |
| *Gss* | glutathione synthetase | NM_008180 |
| *Gstz1* | glutathione transferase zeta 1 (maleylacetoacetate isomerase) | NM_010363 |
| *Mgst1* | microsomal glutathione S-transferase 1 | NM_019946 |
| *Cd68* | CD68 antigen | NM_009853 |
| *Lip1* | lysosomal acid lipase 1 | NM_021460 |
| *Pla2g7* | phospholipase A2 group VII (platelet-activating factor acetylhydrolase, plasma) | NM_013737 |
| *Pla2g1br* | phospholipase A2, group IB, pancreas, receptor | NM_008867 |
| *Plce1* | phospholipase C, epsilon 1 | AF233885 |
| *Pld3* | phospholipase D3 | NM_011116 |
| *Irs1* | insulin receptor substrate 1 | NM_010570 |
| *Acadl* | acetyl-coenzyme A dehydrogenase, long-chain | NM_007381 |
| *Acox1* | acyl-coenzyme A oxidase 1, palmitoyl | NM_015729 |
| *PDX-1* | pancreatic and duodenal homeobox gene 1 | NM_008814 |
| *Irs2* | insulin receptor substrate 2 | XM_976599 |
| *Slc2a2; glut2* | solute carrier family 2 (facilitated glucose transporter), member 2 | NM_031197 |
| *Srebf1; ADD-1; SREBP-1; SREBP-1a; SREBP-1c* | *Mus musculus* mRNA for sterol regulatory element-binding protein-1 (SREBP-1), partial cds | AB017337 |
| *Dgat2* | DAG O-acyltransferase 2 | NM_026384 |
| *F4/80* | *M. musculus* mRNA for F4/80 | X93328 |
| *Ppard* | peroxisome proliferator–activator receptor delta | L28116 |
| *Ppargc1* | peroxisome proliferative–activated receptor, gamma, coactivator 1 | NM_008904 |
| *Ppara* | peroxisome proliferator–activated receptor alpha | NM_011144 |
| *Pparg* | peroxisome proliferator–activated receptor gamma | NM_011146 |
| *Cpt1a* | carnitine palmitoyltransferase 1, liver | AF017175 |
| *G6pc* | glucose-6-phosphatase, catalytic | NM_008061 |
| Glut4 protein | Solute carrier family 2 (facilitated glucose transporter), member 4 *(M. musculus)* | NP_033230 |
| Erk1 protein | Extracellular signal-regulated kinase 1 | Q63844 |
| Erk2 protein | Extracellular signal-regulated kinase 2 | BAA22620 |
